# FOXD1-dependent RalA-ANXA2-Src complex promotes CTC formation in breast cancer

**DOI:** 10.1186/s13046-022-02504-0

**Published:** 2022-10-13

**Authors:** Yufei Long, Tuotuo Chong, Xiaoming Lyu, Lujia Chen, Xiaomin Luo, Oluwasijibomi Damola Faleti, Simin Deng, Fei Wang, Mingliang He, Zhipeng Qian, Hongli Zhao, Wenyan Zhou, Xia Guo, Ceshi Chen, Xin Li

**Affiliations:** 1 Shenzhen Key Laboratory of Viral Oncology, The Clinical Innovation & Research Center (CIRC), Shenzhen Hospital, Southern Medical University, Shenzhen, Guangdong China; 2 The Third School of Clinical Medicine, Southern Medical University, Guangzhou, Guangdong China; 3 Department of laboratory medicine, The Third Affiliated Hospital, Southern Medical University, Guangzhou, Guangdong China; 4 Breast Center, Department of General Surgery, Nanfang Hospital, Southern Medical University, Guangzhou, Guangdong China; 5 Department of Biomedical Sciences, City University of Hong Kong, Hong Kong, China; 6 Guangzhou SaiCheng Bio Co. Ltd, Guangzhou, Guangdong China; 7 Key Laboratory of Animal Models and Human Disease Mechanisms of Chinese Academy of Sciences and Yunnan Province, Kunming Institute of Zoology, Chinese Academy of Sciences Kunming, Kunming, Yunnan China; 8 Academy of Biomedical Engineering, Kunming Medical University, Kunming, Yunnan China; 9 The Third Affiliated Hospital, Kunming Medical University, Kunming, Yunnan China

**Keywords:** Breast cancer, Circulating tumor cells, FOXD1, RalA-ANXA2-Src complex, ERK1/2 inhibitor

## Abstract

**Background:**

Early metastasis is a key factor contributing to poor breast cancer (BC) prognosis. Circulating tumor cells (CTCs) are regarded as the precursor cells of metastasis, which are ultimately responsible for the main cause of death in BC. However, to date molecular mechanisms underlying CTC formation in BC have been insufficiently defined.

**Methods:**

RNA-seq was carried out in primary tissues from early-stage BC patients (with CTCs≥5 and CTCs = 0, respectively) and the validation study was conducted in untreated 80 BC patients. Multiple in vitro and in vivo models were used in functional studies. Luciferase reporter, ChIP-seq, CUT&Tag-seq, and GST-pulldown, etc. were utilized in mechanistic studies. CTCs were counted by the CanPatrol™ CTC classification system or LiquidBiospy™ microfluidic chips. ERK1/2 inhibitor SCH772984 was applied to in vivo treatment.

**Results:**

Highly expressed FOXD1 of primary BC tissues was observed to be significantly associated with increased CTCs in BC patients, particularly in early BC patients. Overexpressing FOXD1 enhanced the migration capability of BC cells, CTC formation and BC metastasis, via facilitating epithelial-mesenchymal transition of tumor cells. Mechanistically, FOXD1 was discovered to induce RalA expression by directly bound to RalA promotor. Then, RalA formed a complex with ANXA2 and Src, promoting the interaction between ANXA2 and Src, thus increasing the phosphorylation (Tyr23) of ANXA2. Inhibiting RalA-GTP form attenuated the interaction between ANXA2 and Src. This cascade culminated in the activation of ERK1/2 signal that enhanced metastatic ability of BC cells. In addition, in vivo treatment with SCH772984, a specific inhibitor of ERK1/2, was used to dramatically inhibit the CTC formation and BC metastasis.

**Conclusion:**

Here, we report a FOXD1-dependent RalA-ANXA2-Src complex that promotes CTC formation via activating ERK1/2 signal in BC. FOXD1 may serve as a prognostic factor in evaluation of BC metastasis risks. This signaling cascade is druggable and effective for overcoming CTC formation from the early stages of BC.

**Supplementary Information:**

The online version contains supplementary material available at 10.1186/s13046-022-02504-0.

## Introduction

Breast cancer (BC) is the leading cause of cancer-associated death in women worldwide [1]. Despite numerous efforts to improve the survival rate of BC in the past decades, about 25–50% of BC patients develop distant metastases after diagnosis [2]. Metastatic breast tumor cells spread to almost all parts of the human body, among which lung, bone, liver, and brain rank as the most frequent metastatic sites [3]. Presently, the five-year survival rate is about 27% in BC patients with recurrence and has not been effectively improved [3–6]. As such, identifying of novel druggable targets for the treatment of BC is crucial.

Circulating tumor cells (CTCs) refer to cancer cells detached from the primary or metastatic tumors and released into blood circulation. They are considered as the ‘precursor event’ of BC metastasis and an important prognostic factor [7, 8]. The American Joint Committee on Cancer (AJCC) TNM staging system incorporated CTC enumeration into a new classification of metastatic staging of BC, and confirmed a significant prognostic effect of CTC count. Several factors, including reactive oxygen species (ROS), tumor cell intravasation, platelets adhesion and epithelial-mesenchymal transition (EMT), etc., have been investigated [9–12], but the molecular mechanisms underlying the regulation of CTC formation in primary BC tissue, particularly in early BC, are scant.

FOXD1 (Forkhead Box D1) is a member of the forkhead gene family of transcription factors and has been implicated in tumorigenesis [13–17]. By activating ALDH1A3 transcription, FOXD1 enhances the oncogenic potential of mesenchymal glioma stem cell-like cells [18]. By regulating MMP9 and RAC1B, FOXD1 promotes invasion in melanoma [19]. By activating the ERK1/2 signal, FOXD1 induces the invasion and metastasis of colorectal cancer [20, 21]. FOXD1 also enhances BC proliferation and chemoresistance [22]. However, to date there has been no evidence linking FOXD1 to CTC formation and metastasis in BC.

Ral (Ras Like) protein is a member of the Ras small G protein family [23]. Activated RalA (GTP-bound RalA) can interact directly with downstream effectors. Oncogenic Ral is often upregulated in various human tumors and plays a key role in oncogenesis and metastasis [24]. ANXA2, an accessory protein that belongs to the calcium-conducting annexin family [25], is highly expressed in breast tumor tissues [26]. Tyr23 Phosphorylation of ANXA2 by Src tyrosine kinase is an important post-translational modification of ANXA2, and p-ANXA2 has significant effects on ERK signal activation, EMT, and metastasis in tumor cells [27–30]. However, little is known regarding the interaction between RalA, ANXA2 and Src in BC.

Here, we carried out a comprehensive study on CTC formation in BC. Clinical sample analyses firstly showed a positive correlation between CTCs in blood samples and FOXD1 expression in primary BC tissue samples, particularly in early BC. High FOXD1 expression was closely associated with increased metastasis and poor outcomes in BC patients. Secondly, comprehensive functional and mechanistic studies provided the first evidence that FOXD1 promoted CTC formation and metastasis in BC by regulating the RalA-ANXA2-Src complex activating ERK1/2 signaling. Further, we conducted an in vivo therapy with ERK inhibitor to evaluate the clinical benefit of FOXD1-dependent cascade on CTC formation and metastasis in BC, providing a potential approach for preventing BC metastasis. Our goal for this study is to give a new perspective on CTC formation and identify novel targets for predicting metastasis risk and preventing metastasis in BC, particularly in early BC.

## Materials and methods

### Clinical samples

Tissue specimens from 80 patients were provided by the department of breast surgery of Nanfang hospital. All BC patients were newly diagnosed and without distant metastasis or clinical treatments. The samples were immediately frozen in liquid nitrogen for RNA extraction and qRT-PCR. The clinical staging of all patients was based on the eighth edition of the AJCC guidelines. 7.5 ml peripheral blood was collected from each patient before treatments, and the circulating tumor cells (CTCs) were counted by the CanPatrol™ CTC classification system (Guangzhou, China). 14 early BC patients were selected for RNA-seq (Novogne, China); among them, 7 patients with CTC ≥ 5 and 7 patients with CTC = 0 (early stage breast cancer was defined as stages I–II [31, 32]). This study was approved by the Ethics Committee of Southern Medical University, and all patients signed informed consent.

### Cell culture

Breast tumor cell lines were obtained from the cell bank of the Chinese Academy of Sciences. MDA-MB-231, MDA-MB-468, T-47D, HS 578 T, and SK-BR-3 were cultured in DMEM containing 10%FBS. MCF-7 cells were cultured in MEM containing 10%FBS and non-essential amino. BT-549 was cultured in 1640 containing 10%FBS. MCF-10A was cultured in MEGM kit (Lonza). 20 mM HEPES, penicillin, and streptomycin were included in all medium formulations. All cells are cultured at 37 °C in a humidified incubator with 5% CO2.

### Total RNA purification

For tissue samples, the tissues were ground with mortar and pestle. After adding TRIzol lysate, the suspension was then homogenized on ice. For the cell samples, TRIzol lysate was added after the adherent cells were washed twice with PBS buffer. After chloroform (200 μl/1 ml TRIzol lysate) was added and mixed, the suspension was placed at room temperature for 5 minutes and centrifuged at 12000 g at 4 °C for 15 minutes. 100-400 μl liquid was pipetted out from the upper layer. Gently mixed it well after adding isopropanol, then centrifuged the suspension at 12000 g at 4 °C for 10 minutes. 75% ethanol (configured with DEPC water and anhydrous ethanol) was added to remove the excess isopropanol reagent. An appropriate amount of DEPC water was added to dissolve the RNA precipitation. Finally, RNA concentration was determined using Nanodrop.

### RT-qPCR

Total RNA was reverse transcribed into cDNA using the RT reagent kit (Takara, RR047A). cDNA was used for subsequent qRT-PCR using the PerfectStart™ Green qPCR SuperMix (Tansgen, AQ602). Briefly, the reagents for gDNA removal were added proportionally, and the mixed liquid was reacted at 42 °C for 2 minutes on a standard PCR instrument. Reverse transcription reaction reagents were proportionally added to the products. The cDNA products were diluted and added to qPCR reaction reagents, including 2X qPCR Supermix, forward and reverse primers (10 μM), nuclease-free water. After lightly blending, a two-step reaction was conducted. According to the results of dissolution and amplification curve analysis, the relative expression of genes was calculated by 2^-△△Ct^. See Supplementary Table 3 for primer information.

### RNA sequencing

After assessing RNA integrity using RNA Nano 6000 (Agilent Technologies, CA, USA). RNA samples of clinical BC tissues or FOXD1 knockdown and control cell lines were subjected to library construction and then sequenced on Illumina Novaseq 6000. FeatureCounts v1.5.0-p3 was used to count the reads numbers mapped to each gene. RNA sequencing technology was provided by Novogene (Beijing, China).

### Zebrafish assay

Two hundred mCherry-labeled cells were suspended in 5 nL DMEM medium for the vitellicle injection of each FLK-EGFP transgenic zebrafish embryo after 48 h fertilization. The numbers of mCherry-labeled cells that intravasated into circulation were observed under CQ1 confocal imaging system (Yokogawa, Japan) and was quantified via ImageJ software.

### Cell counting kit-8 (CCK-8) assay

A total of 1500–2000 cells per well were seeded into 96-well plates. 10 μL of CCK-8 (APExBIO, USA) solution and 100 μL of fresh medium were loaded to each well for incubation at 37 °C for 2 h. The optical density (OD) value was measured at 450 nm using a microplate reader.

### Immunofluorescence

Cells were grown on glass-bottom 24-well culture plates. After treated with 4% paraformaldehyde, the adherent cells were permeabilized with 0.2% Triton X-100. Blocking solution (5% goat serum) was applied for 60 minutes at room temperature. Detailed antibodies information sees Supplementary Table 4. Nuclear staining was performed with DAPI. The images were collected after samples treated with a mounting medium.

### Wound healing assays

5 × 10^5^ cells/well were added to the six-well plate to ensure that the cells were confluent the next day. The next day, a wound was made by dragging a plastic pipette tip across the cell surface. The medium was aspirated, and the floating cells were washed with PBS buffer solution, and the serum-free medium was added to each well. The double turntable high intension confocal was used to record the same position in each well every hour for continuous observation.

### Immunohistochemical staining

Firstly, tissues were fixed in 10% buffered formaldehyde, and then, 3-4 μm paraffin-embedded slides were prepared. After antigen retrieval, the slides were stained with specific primary antibodies and the corresponding species-specific HRP-conjugated secondary antibodies. A Freshly-prepared DAB solution was used for chromogenic reaction, and the nucleus was stained with hematoxylin. Antibodies information see Supplementary Table 4.

### Transwell assays

The cells were resuspended in a serum-free medium, and the cell concentration was adjusted to 10^6 cells/ml. 100 μl/well cell suspension was added in the upper compartment, and 600 μl 10%FBS medium was added in each 24plate well. The residual cells in the upper compartment were cleaned up by swabs after 12-24 h. The cells were fixed with methanol and stained with crystal violet solution.

### Western blot

The total cell protein was extracted by RIPA lysate containing protease/phosphatase inhibitor and denatured. After that, denatured proteins were separated using SDS-PAGE gel, and transferred to the PVDF membrane. The PVDF membrane was incubated with primary antibodies (see Supplementary Table 4) corresponding to the specific protein and species-specific HRP-conjugated secondary antibodies. Blots were visualized by ECL chemiluminescence.

### Cas9-mediated Knock-out of RalA

Disruption of RalA gene in BC cell line MDA231 were adopted from the protocol of our previous work [33]. Two guide RNAs (gRNA1 at 39,686,777) and gRNA2 at 39,687,757) targeting to the genomic locus of RALA NC_000007.14 (39,622,955...39708124, GRCh38.p13) were selected by DNA2.0 /CRISPR online tools. Those guide RNAs combination were introduced to cells to create a big fragment (about 980 bp) deletion around the second exon region of RALA. The transfection was carried out with equimolar mixture of pX459v2-HF-1-RALA_gRNA1/2. 24 hours after post-transfection, the cells were selected with 2 μg/ml puromycin for 3–7 days. At the end of the selection, the puromycin-resistant cells were trypsinized and replated in 96-well at density suitable for single colony isolation of the knockout cell clones. These clonal cell lines were picked from plates and were split into two new replica plates, with one plate for genomic DNA isolation and genotyping PCR, the others for cell expansion till to preservation after validation again by western-blot.

### Dual luciferase reporting assays

RalA promoter (Chr7:39,662,789-39,663,177, hg19) and serial truncations were cloned into pGL4.10[luc2] vector. The indicated reporter vectors were transfected into BC cells together with ectopic expression vector and pGL4.74[hRluc/TK]. Plasmids were transfected with ViaFect™ Transfection Reagent (Promega). After 48 hours cells were harvested. Then, luciferase and Renilla activity were detected according to the manufacture of the Promega dual-luciferase reporter assay kit.

### RalA activation assays

For RalA GTPase pulldowns, RalA activity was measured using RalA Activation Assay Kit (Cat. #BK040, Cytoskeleton, USA). Briefly cell lysates were incubated with RalBP1-RBD beads for 1 hour at 4 °C. After this the beads are washed and boiled in Laemmli Sample Buffer. The boiled samples are run on SDS-PAGE followed by Western Blotting. HRP labeled mouse or rabbit secondary antibodies were used to develop the blots by chemiluminescence using ECL.

### Glutathione S-transferase (GST) pull-down assay

The GST fusion ANXA2 protein (Cat. Ag1777) and 6*His fusion RalA protein (Cat. Ag5329) were purchased from Proteintech™. Briefly, GST or GST-ANXA2 recombinant protein was conjugated to pre-washed anti-GST Tag magnetic beads (Sino Biological, China), followed by the incubation with protein lysates of His-RalA recombinant protein. The immobilized proteins were then eluted and subjected to western blot analysis.

### Co-immunoprecipitation (co-IP) and LC-MS/MS

Co-IP was performed using Pierce Classic Magnetic IP/Co-IP Kit (No.88804, Thermofisher, USA) according to the manufacture’s protocols. Briefly, the total protein of the cells was harvested and incubated with the antibody for FLAG for 12 hours at 4 °C on a rotating wheel. Then, the mixes were incubated together with the protein A/G magnetic beads for 1 hour at room temperature. After extensive washing, the immunocomplexes were eluted and subjected to LC-MS/MS analysis or western blot analysis. For LC-MS/MS analysis, MaxQuant software (version 1.5.6) was applied for protein identification and quantification. According to the LFQ intensity, a protein only identified in FLAG-RalA group with> 2 unique peptides was considered significant. Thus, the top 5 candidates of RalA-interacting proteins were further identified from LC-MS/MS analysis and listed in Supplementary Table 1.

### Chromatin immunoprecipitation (ChIP)

MDA231 cell lines were cross-linked with 1% formaldehyde for 10 min and quenched in 125 mM glycine at RT for 5 min. The formed chromatin was sonicated to generate DNA fragments using Bioruptor plus. Chromatin fragments were immunoprecipitated with antibodies against normal mouse IgG (Invitrogen, USA), FOXD1 (Santa Cruz, USA). Purified DNA was analyzed by qRT-PCR with SYBR Green Master Mix (Transgen, Beijing, China). The primers used are listed in the Supplementary Table 3. ChIP libraries were prepared using ChIP DNA according to the KAPA Hyper Prep Kit library preparation protocol. Libraries were sequenced on Illumina Novaseq 6000.

### CUT&tag

This assay was processed using the NovoNGS CUT&Tag High-Sensitivity Kit V2.0 (Novoprotein, Shanghai) according to the manufacturer’s instruction. 10^6^ cells/sample were harvested and washed in Wash Buffer. ConA magnetic bead-bound cells were resuspended in 50 μL precooled Primary Antibody Buffer containing the appropriate primary antibody (FOXD1, Santa Cruz). Mouse IgG (Invitrogen) was used as the control antibody. After removing the primary antibody, the diluted secondary antibody in 100 μl Secondary Antibody Buffer and cells were incubated at RT for 1 h. ChiTag™ 2.0 Transposome was used to resuspend the cells and the incubation was performed at RT for 1 h. After cells were incubated in Tagmentation Buffer on a rotating platform at 37 °C for 1 h, the DNA were extracted. For libraries amplification, DNA was mixed with i5 Primer, i7 Primer, and 5XAmpliMix were added and mixed, following cycling conditions: 72 °C for 3 min; 98 °C for 30 s; 22 cycles of 98 °C for 15 s, 60 °C for 20 s and 72 °C for 15 sec; final extension at 72 °C for 2 min and hold at 10 °C. Post-PCR clean-up was performed by adding NovoNGS® DNA Clean Beads, and libraries were incubated with beads for 5 min at RT, washed twice gently in 80% ethanol, and eluted in TE-Buffer. Libraries were sequenced by Genefund (Shanghai, China).

### Animal assays

Female-specific pathogen-free (SPF) NSG mice were purchased from Shanghai Model organisms Co.,Ltd. Control or FOXD1-shRNA MDA231 cell was transfected to express luciferase and tdTomato tags. 2 × 10^6^ FOXD1-shRNA (shFOXD1) or control (shCtrl) cells were mixed with 50% Basement Membrane Matrix Phenol Red free (Corning) in PBS and injected orthotopically in mammary fat pad of NSG mice. Primary tumor assessment, CTC enumeration, and lung metastasis analysis were performed 9 weeks after tumor onset. For ERK inhibitor treatment assessment, mice were randomly divided into 5 batches, recorded as batch 1, batch 2, batch 3, batch 4, batch 5. Each batch divided again into 3 experimental groups, CtrL-vehicle, FOXD1-vehicle and FOXD1-SCH (detailed information see Fig. 6 legend). At each observation time point, the CTC number of 3 mice in each group (CtrL-vehicle, FOXD1- vehicle, FOXD1-SCH) were respectively detected. The mice of batch 5 were continuously imaged by BLI (Fig. 6D). In detail, 2 × 10^6^ MDA231-FOXD1-Luc-tdTomato or MDA231-FOXD1-CtrL-Luc-tdTomato cells were mixed with 50% Basement Membrane Matrix Phenol Red free (Corning) in PBS and injected orthotopically in NSG mice. When tumor volumes reached ≈50mm^3^, SCH772984 (25 mg/kg, formulated in DMSO) or vehicle (DMSO) was intraperitoneally injected twice per day for 2 weeks. All animal experiments were performed according to the university animal guidelines, and with prior approval from the Animal Experimentations Ethics Committee, Southern Medical University.

### CTC capture and identification

Peripheral blood specimens for CTC analysis were obtained after informed patient consent at Nanfang Hospital. 7.5 ml of peripheral blood was drawn in EDTA vacutainers. The first 2 ml were discard to avoid potential skin cell contamination from the venipuncture. The CanPatrol™ system (Guangzhou, China) was used to isolate CTCs as previously described [34]. Briefly, the prepared blood samples were filtrated through a membrane with 8-μm diameter pores. The CTCs were retained on the filter, and the leukocytes went through the pores because CTCs were larger than leukocytes. Then, the CTCs were identified by RNA-ISH. Briefly, the cells retained on the filter were fixed, permeabilized and digested. Next, the cells were subjected to a serial of hybridization reactions with probes specific to the EpCAM, CK8/18/19, Vimentin, Twist, and CD45 transcripts. DAPI was used to stain the nucleuses. The cells were analyzed with a fluorescent microscope. The CTCs were identified by probes specific for the genes mentioned above (EpCAM, CK8/18/19, Vimentin and Twist), and CD45 was used to discriminate leukocytes and CTCs. For mouse studies, the CTCs were captured by microfluidic immunomagnetic bead screening provided by LiquidBiospy™ CTCs enrichment and detection platform (Livzon Cynvenio) [35], according to the manufacturer’s instructions. Briefly, the blood samples were retrieved via cardiac puncture. The prepared blood samples were added with biotin-labeled capture antibodies (including EpCAM, and EMT relative antibodies), streptavidin-labeled magnetic beads. In addition, fluorescence-labeled CD45 antibody was added to detect and eliminated leukocytes. Nucleuses were stained by DAPI. The enriched CTCs on microfluidic chips were identified and counted by Ariol DM6000B system (Leica Microsystems). The CTCs were defined as nucleated cells lacking CD45 and expressing eGFP.

### Statistical analyses

All data were extracted from not less than three independent experiments. To ascertain statistical differences between two groups or multiple testing, Student’s t-test or One-way ANOVA was used ascertain posttest combined with Tukey test used to compare all pairs of groups. The χ2 or Fisher exact tests were used for categorical variables. Correlation analysis was performed using Pearson’s correlation. *P* < 0.05 was considered significant.

## Results

### FOXD1 is clinically involved in CTC formation in BC, particularly in early BC

**Fig. 1:**
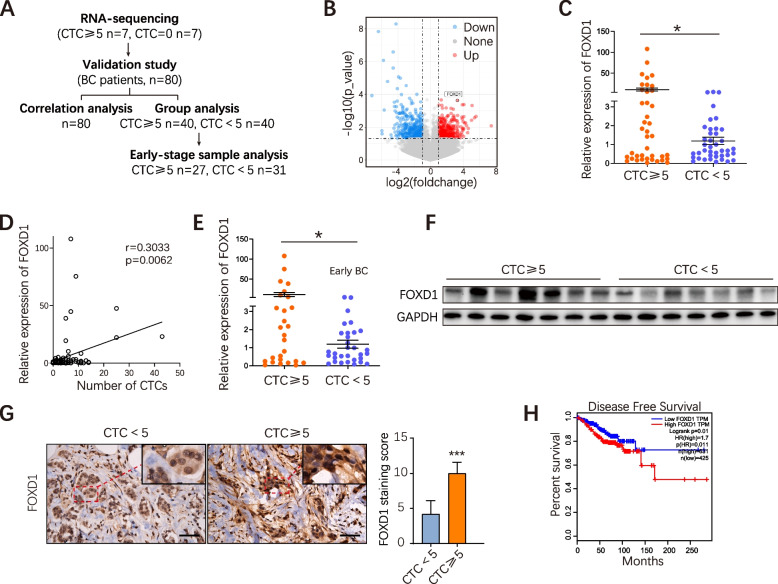
FOXD1 is clinically involved in CTC formation in BC, particularly in early BC. **A** Analysis workflow of clinical BC tumor samples. **B** Volcano plot showing the differential RNA expression genes (by RNA-seq) between clinical BC primary tumors with CTC ≥ 5 and clinical BC primary tumors with CTC = 0. *n* = 7. **C** The mRNA levels of FOXD1 in the BC primary tissues with CTC ≥ 5 or CTC<5 were analyzed by qRT-PCR. Error bars, SEM. CTC ≥ 5, *n* = 40; CTC<5, *n* = 40. *p<0.05 by Student’s t-test. **D** Correlation between CTC number in BC patients and FOXD1 expression in primary tumor. *n* = 80. **E** FOXD1 mRNA levels in primary tissues of early-stage BC patients were analyzed by qRT-PCR. Error bars, SEM. CTC ≥ 5, *n* = 27; CTC<5, *n* = 31. *p<0.05 by Student’s t-test. **F** Western blotting analyses of FOXD1 expression in primary tumors with CTC ≥ 5 or CTC<5. GAPDH as a control gene. **G** Immunohistochemistry staining (IHC) of FOXD1 in indicated BC patient primary tumors. Scale bar, 50 μm. Error bars, SD. *n* = 5. ***p < 0.001 by Student’s t-test. **H** Disease-free survival analysis of BC patients with high FOXD1 expression by TCGA database

A total of 80 patients who had been clinically diagnosed with BC at Nanfang hospital, Southern Medical University, China, were enrolled in this study. Using the CanPatrol™ CTC classification system (Guangzhou, China) [34], CTCs were isolated from peripheral blood samples (7.5 ml) collected prior to the commencement of treatment. According to the 8th edition of the American Joint Committee on Cancer (AJCC) TNM staging system, CTC ≥ 5 /7.5 mL in peripheral blood of BC patients indicates poor prognosis. We randomly selected 7 patients with CTCs≥5 and 7 patients with CTCs = 0 (Fig. 1A). All patients had early-stage BC with similar primary foci size. Comparing the gene expression profiles of primary BC tissues derived from these two groups, we obtained a list of 103 differentially expressed genes (37 were upregulated and 66 were down-regulated) (Fig. 1B). According to the *p*-value and foldchange (Fig. 1B), we paid attention to FOXD1, which was previously implicated in cell proliferation and chemoresistance in BC [22] and its exact role in CTC formation has not been reported in BC so far. Notably, the verification of FOXD1 differential expression in a second cohort (CTCs≥5, *n* = 40 and CTCs<5, n = 40) showed a similar expression trend of FOXD1 to sequencing data (Fig. 1C). FOXD1 mRNA expression in primary foci positively correlated with CTCs in peripheral blood (*R* = 0.3033, *P* = 0.0062) (Fig. 1D). FOXD1 protein expression level in the CTCs≥5 group was significantly higher than the CTCs<5 group (Fig. 1F-G). Moreover, it was particularly noteworthy that, in the early BC patients, FOXD1 mRNA expression still remained higher in CTCs≥5 group than in CTCs<5 group (Fig. 1E), suggesting that FOXD1 was implicated in CTC formation in BC, particularly in early BC. The Cancer Genome Atlas (TCGA) database revealed that FOXD1 was highly expressed in a variety of other tumors (Supplementary Fig. 2E). The TCGA database also supported that the disease-free survival was significantly low in the BC patients with high FOXD1 expression relative to the BC patients with low FOXD1 expression (Fig. 1H).

Together, these results indicate that FOXD1 is clinically correlated with CTC formation in BC, particularly in early BC.

### FOXD1 promotes CTC formation in BC via promoting EMT

We initially investigated six BC cell lines and normal breast epithelial cell line MCF-10A. Compared with MCF-10A, six BC cells showed higher expression levels of FOXD1 (Supplementary Fig. 1A-B). We designed lentivirus vectors carrying FOXD1 short hairpin RNA (shRNA) sequences (sh1, sh2, sh3) and infected two of six cell lines, MDA-MB-231 (MDA231) and MDA-MB-468 (MDA468), with relatively high endogenous FOXD1 expression. shRNA effectively knocked down the FOXD1 expression in both cells at the mRNA level and protein level (Supplementary Fig. 1C-D). We also established FOXD1-overexpressing MCF-7 cells, which originally had a relatively low FOXD1 expression (Supplementary Fig. 1C-D).

**Fig. 2:**
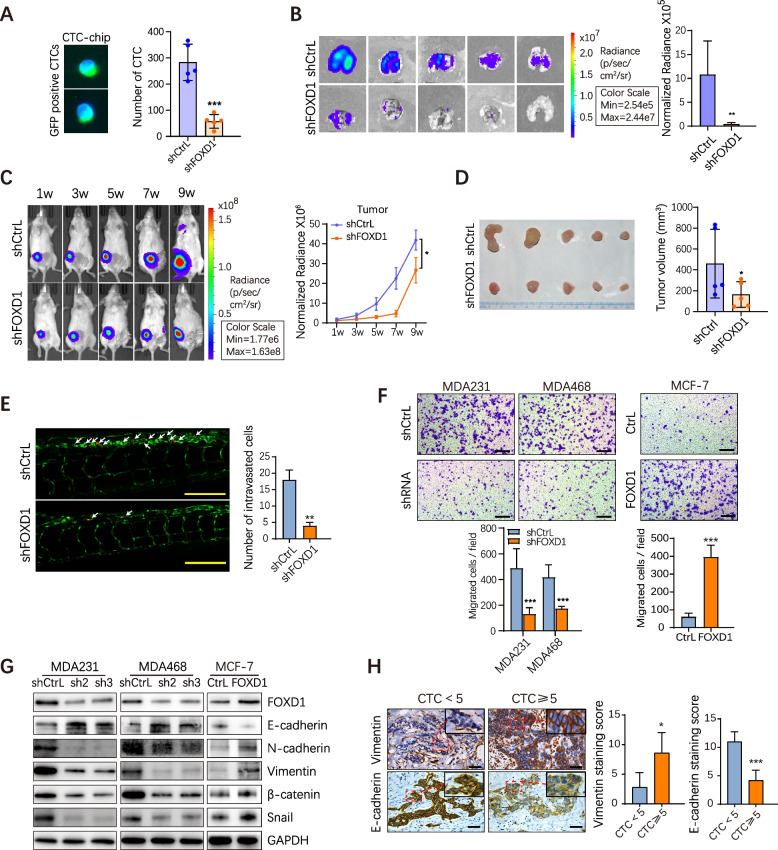
FOXD1 promotes the CTC formation in BC via regulating EMT. **A** Bar graphs showing the CTC number from mice bearing a MDA231 control or FOXD1 knockdown primary tumor at week 9. The CTCs were captured by the microfluidic CTC chip. Error bars, SD. *n* = 5. ***p < 0.001 by Student’s t-test. **B** Images of bioluminescence imaging (BLI) of the lung from two indicated groups at week 9. Bar graph showing normalized lung photons detected by BLI. Error bars, SEM. *n* = 5. **p<0.01 by Mann-Whitney test. **C** Representative images of BLI of mice on 1, 3, 5, 7, 9 weeks after orthotopic injection of indicated cells. Bar graphs showing normalized tumor photons from two indicated groups. Error bars, SEM. *n* = 5. *p<0.05 by Student’s t-test. **D** Representative images of the xenograft tumors isolated from two indicated groups at week 9. The tumor volumes of xenograft tumors isolated from shCtrL and shFOXD1 groups were analyzed by bar graph. Error bars, SEM. *n* = 5. *p<0.05 by Student’s t-test. **E** Representative images of zebrafish from two indicated groups. The numbers of control or FOXD1 knockdown cells (white arrows indicated) intravasated into circulation were showed by bar graph. Scale bar, 200 μm. Error bars, SD. *n* = 3. **p < 0.01 by Student’s t-test. **F** Cell migration capacity of FOXD1 knockdown cells (MDA231 and MDA468) or FOXD1 overexpression cells (MCF7) and control cells was determined by the transwell. Representative photographs of migratory cells are shown. Scale bar, 250 μm. Error bars, SD. *n* = 3. ***p < 0.001 by Student’s t-test. **G** Expression of EMT relative proteins (E-cadherin, Vimentin, N-cadherin, β-catenin, Snail) in CtrL and FOXD1 knockdown cells (MDA231 and MDA468), Ctrl and FOXD1 overexpression cells (MCF-7) were analyzed by Western blot. **H** IHC analyzed E-cadherin and Vimentin expression in primary tumors with CTC ≥ 5 or CTC<5. Scale bar, 50 μm. Error bars, SD. *n* = 5. *p<0.05, ***p < 0.001 by Student’s t-test

To elucidate the function of FOXD1 in regulating CTC formation, we established immunodeficient (NSG) mice bearing MDA231-shFOXD1-Luc and MDA231-shCtrL-Luc cells. Blood samples (about 0.8-1 ml) were collected from mice. CTCs were enriched by LiquidBiospy™ CTCs enrichment and detection platform (Livzon Cynvenio) [35]. We noticed that the number of CTCs was significantly lower in FOXD1 knockdown group than in control group (Fig. 2A). Metastatic bioluminescence signals from lungs were significantly lower in the FOXD1 knockdown group than the control (Fig. 2B). Similarly, tumor growth rate (Fig. 2C) and tumor volume (Fig. 2D) were reduced in mice bearing FOXD1-knockdown cells. Moreover, Zebrafish metastatic xenograft model revealed that the metastatic cells (marked by white arrows) in the tail of zebrafish injected with FOXD1 knockdown MDA231 cells were fewer than that in the control group (Fig. 2E). In consistence with in vivo results, knockdown of FOXD1 decreased the migration potential of BC cells, as shown by transwell and wound healing experiments, while overexpression of FOXD1 showed opposite effects (Fig. 2F and Supplementary Fig. 2A).

EMT plays an essential role in BC metastasis and CTC formation [36–38]. Immunoblot analyses showed that, in the FOXD1 knockdown group, the expression of E-cadherin was increased while the expression of N-cadherin, Vimentin, Snail and β-catenin was decreased. Inversely, stable overexpression of FOXD1 decreased E-cadherin expression and increased the expression of N-cadherin, Vimentin, Snail and β-catenin (Fig. 2G and Supplementary Fig. 2B). We also observed morphologic changes in MCF-7-FOXD1 cells from circular or polygonal to long spindle shape, suggesting the existence of EMT-associated phenotypic features (Supplementary Fig. 2C). We further detected the expression of EMT genes in primary tissues of clinical BC patients. Consistently, BC patients with CTCs≥5 had higher Vimentin expression and lower E-cadherin expression than the CTCs<5 group (Fig. 2H). The expression trend of Vimentin protein was consistent with that of FOXD1 (Fig. 2H and Fig. 1G). We also investigated the effects of FOXD1 on cell proliferation. CCK-8 assays showed that FOXD1 increased BC cell growth (Supplementary Fig. 2D).

Collectively, these findings demonstrate that FOXD1 may promote the development of CTCs and metastasis in BC by inducing EMT.

### FOXD1 activates the ERK1/2 signal by regulating RalA in BC cells

**Fig. 3:**
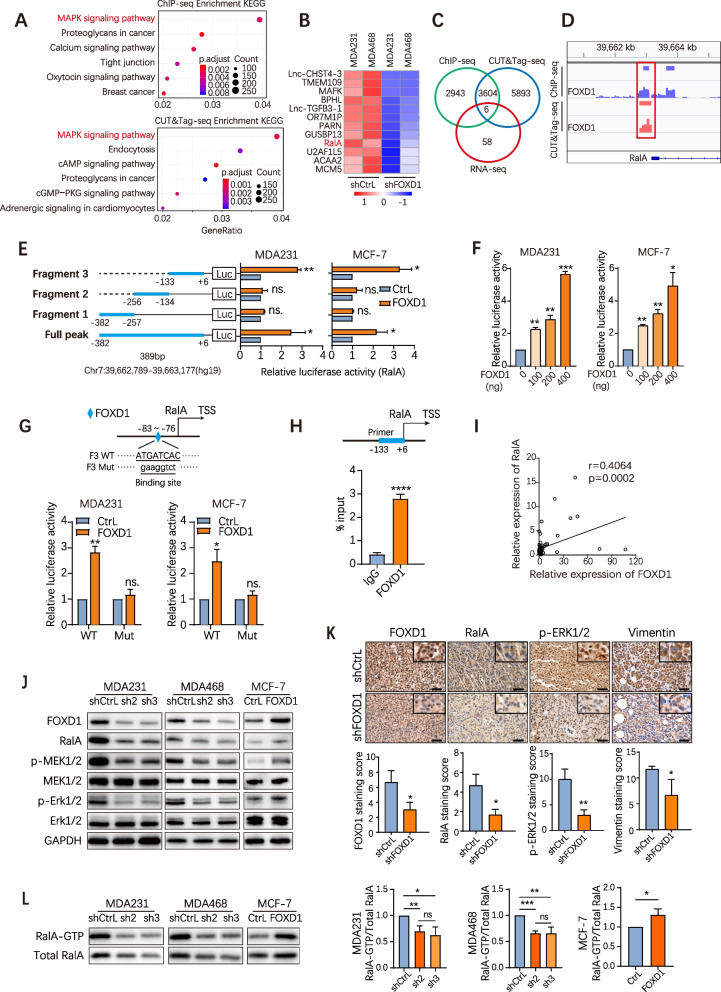
FOXD1 activates the ERK1/2 signal by regulating RalA in BC cells. **A** KEGG analysis of the FOXD1 binding sites detected by ChIP-seq (up panel) and CUT&Tag-seq (down panel). **B** Heat map showing RNA differential expression genes (by RNA-seq) between control and FOXD1 knockdown cells. The genes were identified with significant expression change and fold change > 1.5. **C** Venn diagram of FOXD1 target selection. For both ChIP-seq and CUT&Tag-seq analysis, the genes with FOXD1 promotor binding in − 1000 ~ 0 bp to TSS were selected. **D** Representative images of ChIP-seq and CUT&Tag-seq results with FOXD1 antibody on RalA genomic region. RalA-peak represents the DNA region that was cloned and used in (E). **E** Dual luciferase reporter assay with FOXD1 overexpression. Schematic view of FOXD1 binding region on RalA TSS. Truncations were ligated to pGL4.10[Luc2] vector. Dual luciferase assay was performed with co-transfection of pEXP-FOXD1 and full-length or truncated reporter vectors. Error bars, SD. *n* = 3. *p < 0.05, **p < 0.01 by Student’s t-test. ns. = not significant. **F** Dual luciferase assays of MDA231 cells or MCF-7 co-transfected with a RalA-fragment 3 (F3) promoter-reporter plasmid and overexpression vectors for FOXD1 (gradient dilution). Error bars, SD. *n* = 3. *p < 0.05, **p < 0.01, ***p < 0.001 by Student’s t-test. **G** Dual luciferase assays demonstrated the expression of RalA-F3 (WT or mutant form) by MDA231 or MCF-7 cells transfected with pEXP-FOXD1 or with control. Error bars, SD. *n* = 3. *p < 0.05, **p < 0.01 by Student’s t-test. ns. =not significant. **H** ChIP-qPCR assay using FOXD1 antibody or control IgG in MDA231 shows the binding of FOXD1 on the RalA promoter. Error bars, SD. *n* = 3. ****p < 0.0001 by Student’s t-test. **I** Association of RalA expression with FOXD1 expression at clinical BC primary tumors. *n* = 80. **J** Western blot analyses of RalA and MAPK pathway relative protein expression in CtrL and FOXD1 knockdown cells (MDA231 and MDA468), Ctrl and FOXD1 overexpression cells (MCF-7). GAPDH served as a loading control. **K** IHC analyzed the expression of FOXD1, RalA, p-ERK1/2, and Vimentin in xenograft tumors from mouse models. Scale bar, 50 μm. Error bars, SD. *n* = 3. *p<0.5, **p<0.01 by Student’s t-test. **L** RalA activation assays in the indicated cells. Bar graphs showing quantification of GTP-RalA/Total-RalA ratio. Error bars, SD. *n* = 3. *p< 0.5, **p< 0.01 and ***p< 0.001 by Student’s t-test

To identify the molecules directly regulated by FOXD1 in CTC formation, we performed ChIP-sequencing and CUT&Tag-sequencing analyses. Gene enrichment pathway (KEGG) analyses revealed that FOXD1 downstream genes were preferentially enriched in the top-ranked MAPK signaling pathway (Fig. 3A). We also investigated the global transcriptional alteration (RNA-seq) caused by FOXD1 in two pairs of stable FOXD1 knockdown cells (MDA231-shFOXD1, MDA468-shFOXD1) and their corresponding control cells (Fig. 3B). Next, using Venn diagram analysis for three datasets from RNA-seq, ChIP-seq and CUT&tag-seq, we discovered 6 common genes, of which RalA (a Ras-like small GTPase) was identified as a FOXD1 downstream target gene uniquely associated with the MAPK pathway (Fig. 3C). Results from ChIP-seq and CUT&Tag-seq analyses showed that FOXD1 bound to the upstream (− 382 ~ + 6 bp, chr7:39,662,789 -39,663,177) of the transcription start site (TSS) of RalA (Fig. 3D).

To confirm the ChIP-seq and CUT&Tag-seq results, luciferase reporters were constructed by fusing the enriched peak region of RalA promoter with the luciferase gene (Fig. 3E). We found that the ectopic expression of FOXD1 majorly increased the luciferase reporter activity of RalA-Fragment 3 (F3) compared to the controls (Fig. 3E-F and Supplementary Fig. 3A). To further confirm the FOXD1 binding site within the F3 of RalA promoter, mutant constructs for FOXD1 binding sites (− 83 ~ − 76 bp) were generated after using analysis of JASPAR database (Additional file 2). The enhanced luciferase activity was reversed by transfection with mutant promoter region (Fig. 3G). Moreover, the transcriptional regulation of RalA by FOXD1 was supported by ChIP-qPCR, which indicated that FOXD1 directly bound to the promoter region of RalA in BC cells (Fig. 3H). In the clinical primary BC tissues, RalA expression level was also positively correlated with FOXD1 (*R* = 0.4064, *P* = 0.0002) (Fig. 3I).

Furthermore, western blot showed that knockdown of FOXD1 markedly reduced RalA and the phosphorylation of MEK1/2 and ERK1/2 (MAPK pathway proteins) while the expression of non-phosphorylated proteins remained unchanged. Overexpression of FOXD1 upregulated RalA and the phosphorylation of MEK1/2 and ERK1/2 (p-MEK1/2, p-ERK1/2) (Fig. 3J). Immunohistochemical staining of primary mouse tissues indicated that the expression of RalA, p-ERK1/2 and Vimentin in the FOXD1 knockdown group were lower than those in the control group (Fig. 3K). Together, these data indicated that FOXD1 majorly influenced the MAPK signaling pathway in BC cells.

It is known that RalA is a small Ras-related protein that functions as a GTPase, so we further examined whether FOXD1 influenced RalA GTPase activity. Interestingly, we observed that RalA GTPase activity was significantly reduced upon the inhibition of FOXD1 expression. In contrast, GTPase activity was enhanced after FOXD1 was overexpressed (Fig. 3L).

Taken together, these findings support that FOXD1 transcriptionally regulates RalA and enhances its GTPase activity in BC cells.

### FOXD1-RalA-ERK1/2 signaling cascade mediates CTC formation and BC cell migration

To determine whether the CTC formation and BC cell migration were regulated by FOXD1-RalA-ERK1/2 signaling cascade, we performed CRISPR/Cas9-mediated knockout of RalA in MDA231 cells. RalA knockout reduced the migration ability of BC cells (Supplementary Fig. 3E-G), down-regulated ERK1/2 signal, and impaired EMT process (Supplementary Fig. 3I). The cell growth ability of BC cells also reduced after RalA knockout (Supplementary Fig. 3H).

**Fig. 4:**
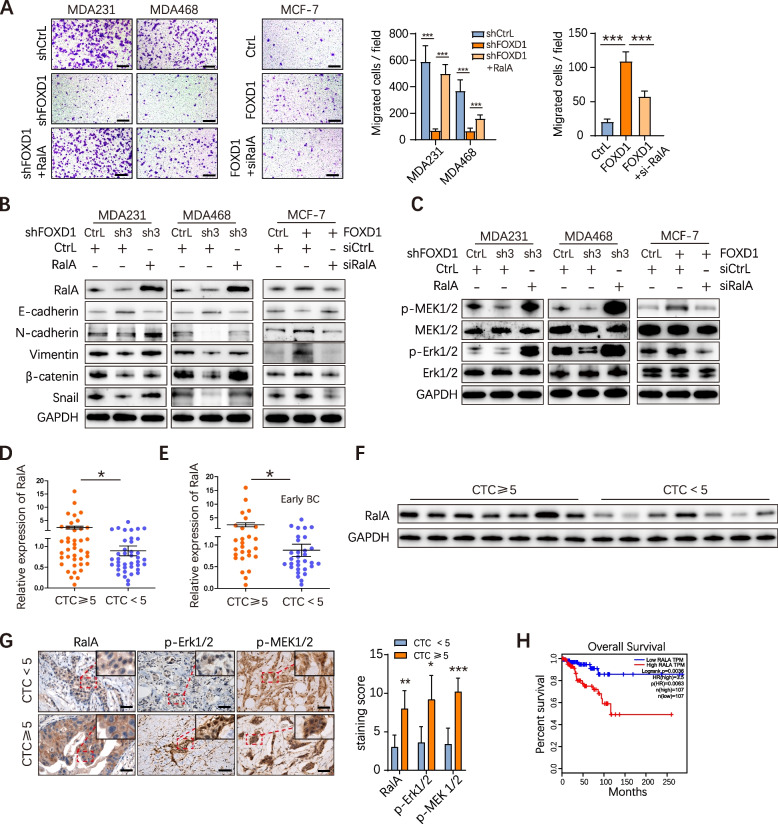
FOXD1-RalA-ERK1/2 signaling cascade mediates CTC formation and cell migration. **A** BC cells (MDA231 and MDA468) with FOXD1 knockdown alone or in combination with RalA ﻿were subjected to transwell migration assays. MCF-7 with FOXD1 overexpression alone or in combination with si-RalA ﻿were subjected to transwell migration assays. Scale bar, 250 μm. Error bars, SEM. *n* = 3. ***p<0.001 by Student’s t-test. **B, C** EMT relative markers (E-cadherin, N-cadherin, Vimentin, Snail, β-catenin) and MAPK pathway relative proteins expression levels were measured via western blotting. GAPDH detection was shown as a control. **D** The mRNA levels of RalA in the clinical BC primary tissues with CTC ≥ 5 or CTC<5 were analyzed by qRT-PCR. Error bars, SEM. CTC ≥ 5, *n* = 40; CTC<5, *n* = 40. *p < 0.05 by Student’s t-test. **E** RalA mRNA levels in clinical BC primary tissues of early-stage BC patients were analyzed by qRT-PCR. Error bars, SEM. CTC ≥ 5, *n* = 27; CTC<5, *n* = 31. *p<0.05 by Mann-Whitney test. **F** Western blotting analyses of RalA expression in clinical BC primary tumors with CTC ≥ 5 or CTC<5. GAPDH detection is shown as a control. **G** IHC analyzed RalA, p-ERK1/2 and p-MEK1/2 expression in clinical BC primary tumors with CTC ≥ 5 or CTC<5. Scale bar, 50 μm. Error bars, SD. *n* = 5. *p < 0.05, **p < 0.01 by Student’s t-test. **H** Overall survival analysis of BC patient with high RalA expression, by TCGA database

Subsequently, RalA rescue experiments were performed. After overexpressing RalA, the reduction of cell migration ability caused by FOXD1 knockdown was observed to be largely restored (Fig. 4A), and the expression of E-cadherin was downregulated while the expression of p-MEK1/2, p-ERK1/2, N-cadherin, Vimentin, β-catenin and Snail was upregulated (Fig. 4B-C and Supplementary Fig. 3B). RalA also partially restored the reduced cell growth ability caused by FOXD1 knockdown (Supplementary Fig. 3 J). Moreover, we used siRNA to suppress RalA expression in MCF-7-FOXD1 cells. Knockdown of RalA significantly reduced the cell migration of MCF-7-FOXD1 cells (Fig. 4A), upregulated E-cadherin expression and down-regulated the expression of p-MEK1/2, p-ERK1/2, N-cadherin, Vimentin, β-catenin and Snail (Fig. 4B-C and Supplementary Fig. 3C). CCK-8 assay also showed that the proliferation of MCF-7-FOXD1 cells was significantly reduced upon RalA knockdown (Supplementary Fig. 3 J).

SCH772984 is a preclinically promising, selective, ATP-competitive ERK1/2 inhibitor that inhibits the phosphorylation of the ERK self-activating ring [39, 40] (Supplementary Fig. 4A and Supplementary Fig. 3D). Using ERK inhibitor, we continued to testify the regulatory role of ERK1/2 signal in FOXD1’s promotion of BC cell migration. We found that SCH772984 attenuated the migration ability of MDA231-FOXD1 cells in a dose-dependent manner (Supplementary Fig. 4B). It also significantly impaired EMT process in MDA231-FOXD1 cells (Supplementary Fig. 4C). CCK-8 assays also showed that the SCH772984 reduced the proliferation of FOXD1-overexpressing tumor cells (Supplementary Fig. 4D).

Furthermore, we clinically assessed the correlation between RalA and CTCs in BC patients. RT-qPCR showed that RalA expression level in primary BC tissues with CTCs≥5 (*n* = 40) was significantly higher than that in primary BC tissues with CTCs<5 (*n* = 40) (Fig. 4D). Notably, consistently with that of FOXD1 expression, it is in the early BC patients that RalA expression was still higher in the CTCs≥5 group (*n* = 27) than in the CTCs<5 group (*n* = 31), suggesting that RalA also played a role in CTC formation in the early BC patients (Fig. 4E). Moreover, Western blot and IHC results showed that the expression level of RalA in the group with CTCs≥5 was higher than that in the group with CTCs<5 (Fig. 4F-G). The TCGA database indicated that the overall survival of BC patients with high RalA expression was significantly shorter than that of patients with low RalA expression (Fig. 4H). Finally, IHC analysis showed that the expression levels of p-ERK1/2 and p-MEK1/2 were higher in primary tissues with high CTCs than those of BC patients with low CTCs (Fig. 4G).

Collectively, the above results demonstrate that the FOXD1-dependent RalA-ERK1/2 signaling cascade mediates CTC formation and BC cell migration.

### RalA-ANXA2-Src complex is required for activating ERK1/2 signal

To gain further insight into the mechanisms underlying the activation of ERK1/2 signal by RalA, we applied immunoprecipitation-LC-MS to identify the potential binding partners of RalA (Additional file 3). Interestingly, Annexin A2 (ANXA2) was identified as a major protein partner of RalA (Supplementary Table 1).

**Fig. 5:**
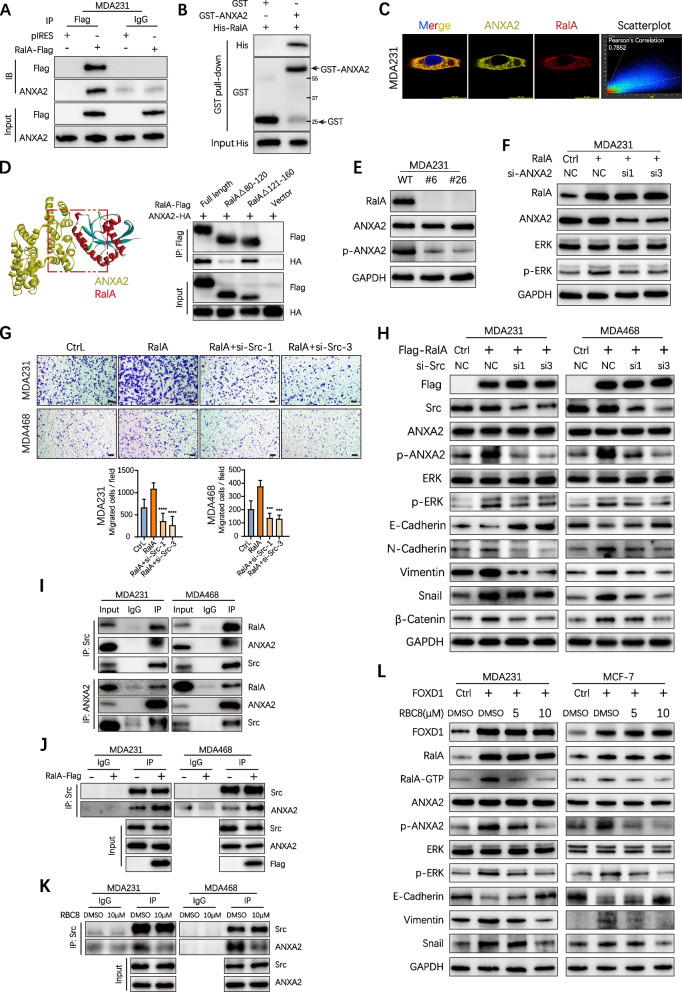
RalA-ANXA2-Src complex is required for activating ERK1/2 signal. **A** The interaction between RalA and ANXA2 was confirmed by co-immunoprecipitation in MDA231 cells with transient RalA overexpression. **B** In vitro interactions between RalA and ANXA2 was determined by GST pull-down assay. **C** Confocal staining presented the co-localization of RalA and ANXA2 in MDA231 cells. Pearson’s correlation (0.7852) of the co-localization between RalA and ANXA2 was analyzed by Las X software. **D** ZDOCK used to predict the binding sites of RalA and ANXA2. Constructs with deleted mutations Δ80–120 and Δ121–160 of RalA-Flag were used for immunoprecipitation with ANXA2-HA. **E** WT and RalA KO MDA231 cells were subjected to immunoblotting assays. **F** Western blot analyses of ERK1/2 and p-ERK1/2 in MDA231 cells transduced with si-ANXA2 or ctrl. GAPDH serves as a loading control. **G** BC cells with RalA overexpression alone or in combination with si-Src were subjected to transwell migration assays. Scale bar, 100 μm. Error bars, SEM. *n* = 3. ***p<0.001, ****p < 0.0001 by Student’s t-test. **H** RalA overexpression MDA231 and MDA468 cells in combination with si-Src were subjected to western blotting assays. **I** Co-immunoprecipitation assays showed that endogenous Src interacted with endogenous RalA and ANXA2 in BC cells. Endogenous ANXA2 interacted with endogenous RalA and Src in BC cells. **J** Co-immunoprecipitation assay showed the interaction between ANXA2 and Src in control or RalA overexpression BC cells. **K** The interaction between ANXA2 and Src in control (DMSO) or RBC8 (Selleck, s7606) treated BC cells were verified by co-immunoprecipitation assays. **L** FOXD1 overexpression MDA231 and MCF7 cells in combination with RBC8 (0, 5, and 10 μM, 48 h) were subjected to western blotting assays. RalA-GTP levels were analyzed by pulldown assays

Thus, we focused our subsequent studies on the role of ANXA2 in RalA-dependent activation of ERK1/2 signal. The interaction between RalA and ANXA2 was confirmed by co-immunoprecipitation (co-IP) in MDA231 cells and GST-pulldown (Fig. 5A-B). The co-localization of RalA and ANXA2 was observed in the cytoplasm using immunofluorescent staining (Fig. 5C). To further ascertain the crucial binding site of RalA, ZDOCK was used to build an interaction model of these two proteins (Supplementary Table 2). Co-IP assay confirmed that aa80–120 of RalA was necessary for the direct interaction between RalA and ANXA2 (Fig. 5D). Notably, CRISPR/Cas9-mediated knockout of RalA was found to reduce the phosphorylation of ANXA2 (p-ANXA2, Tyr23), but did not obviously alter total ANXA2 (Fig. 5E). These data suggested that RalA formed a complex with ANXA2, inducing the phosphorylation of ANXA2.

ANXA2 belongs to the calcium-conducting annexin family, which has been reported to activate MAPK signaling and promote metastasis in tumor cells. Tyrosine phosphorylation (Tyr23) of ANXA2 was required for ERK1/2 activation [30, 41, 42]. In RalA-overexpressing MDA231 cells, we observed that ANXA2 knockdown indeed reduce p-ERK1/2 (Fig. 5F), confirming that ANXA2 was required for RalA-dependent activation of ERK1/2 signal.

ANXA2 was first described as a substrate for the Src kinase and previously reported to bind to Src [43, 44]. To explore whether Src kinase is involved in the phosphorylation of ANXA2, we used si-Src in MDA231-RalA and MDA468-RalA cells and found that si-Src reduced the cell migration ability (Fig. 5G) and down-regulated the expression of p-ANXA2 (Fig. 5H). Given that RalA was interacted with ANXA2 and ANXA2 was reported to bind to Src [44], we proposed that RalA may form a complex with ANXA2 and Src in BC cells. Notably, co-IP assays showed that Src co-precipitated with RalA and ANXA2, while ANXA2 interacted with RalA and Src in an endogenous condition (Fig. 5I). Also, we predicted the model of RalA-ANXA2-Src ternary interaction (Supplementary Fig. 4E). RalA overexpression conferred no evident effect on the expression levels of Src or ANXA2, whereas the interaction between Src and ANXA2 was notably enhanced compared with control cells (Fig. 5J), suggesting that RalA was required for the binding of Src to ANXA2. In addition, Ral GTPase inhibitor RBC8 was used to test whether RalA-GTP activity was necessary for the interaction between ANXA2 and Src. Co-IP assay showed that the binding ability of ANXA2 to Src was decreased in RalA-GTP inhibited cells compared with control cells (Fig. 5K). Using RBC8 obviously reduced the expression of p-ANXA2, p-ERK1/2 and the EMT process in FOXD1 overexpression cells (Fig. 5L). Moreover, ectopic expression of constituently activated RalA mutant (G23V) induced p-ERK1/2, whereas constituently inactivated RalA mutant (G26A) decreased p-ERK1/2 level, indicating RalA-GTP indeed activated ERK1/2 signaling pathway (Supplementary Fig. 4F). These data collectively suggest that RalA forms a complex with ANXA2 and Src, mediates the interaction between Src and ANXA2, and thus promotes the phosphorylation of ANXA2. This RalA-ANXA2-Src complex is essential for activating ERK1/2 signaling cascade and promoting metastasis ability of BC cells.

### In vivo blockade of ERK1/2 with SCH772984 reduces CTC number and BC metastasis

**Fig. 6:**
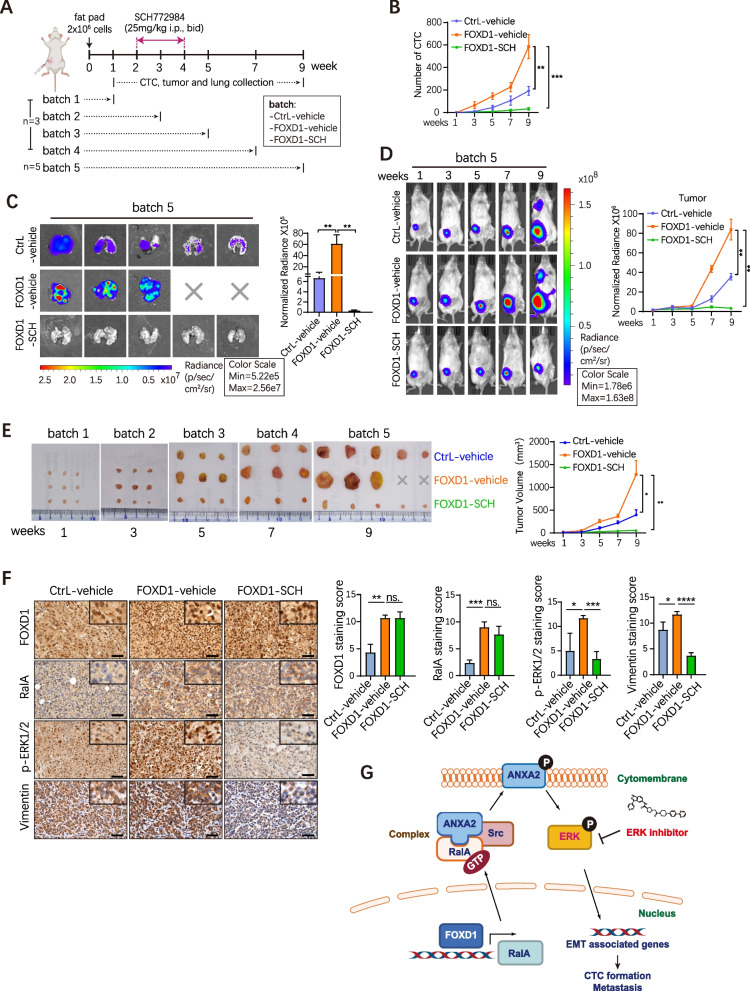
In vivo Blockade of ERK1/2 with SCH772984 reduces CTC number and BC metastasis. Note: Mice were randomly divided into 5 batches, recorded as batch 1, batch 2, batch 3, batch 4, batch 5. Each batch divided again into 3 experimental groups, CtrL-vehicle, FOXD1-vehicle and FOXD1-SCH. The observation times of 5 batches were 1, 3, 5, 7, 9 weeks respectively. For batch 1–4, CTC capture and xenograft tumors isolation analyses were performed on each mouse (3 mice/group). For batch 5, continuously BLI (from week 1 to 9), CTC capture, xenograft tumors isolation and BLI of lungs analyses were performed on each mouse (5 mice/group). **A** Schematic view of SCH772984 treatment xenograft model. Control or FOXD1 overexpression MDA231 cells were injected into mammary fat pad. After the tumor volume reached approximately 50 mm3, SCH772984 were diluted in DMSO and were delivered through intraperitoneal injection twice a day with a concentration of 25 mg/kg for each mouse. **B** The CTC numbers of the indicated group at each time point. At each observation time point, the CTC number of 3 mice in each group (CtrL-vehicle, FOXD1- vehicle, FOXD1-SCH) were respectively detected. The blood samples were retrieved via cardiac puncture. Error bars, SEM. *n* = 3. **p<0.01, ***p < 0.001 by Student’s t-test. **C** BLI of lungs at the last observation time point (batch 5). Error bars, SEM. **p<0.01 by Student’s t-test. **D** BLI was continuously performed on the mice of batch 5. Representative images of BLI at week 1, 3, 5, 7, 9 after control or FOXD1 overexpression MDA231 cells were injected into mammary fat pad. Error bars, SEM. **p < 0.01 by Student’s t-test. **E** Images of all xenograft tumors isolated from each indicated batch. Error bars, SEM. *p<0.05, **p < 0.01 by Student’s t-test. **F** IHC analyzed the expression FOXD1, RalA, p-ERK1/2, and Vimentin in the xenograft tumor from mouse models at the last time point (week 9). Scale bar, 50 μm. Error bars, SD. *n* = 3. *p<0.05, **p < 0.01, ***p < 0.001 by Student’s t test. ns. = not significant. **G** A schematic model of the FOXD1-dependent RalA-ANXA2-Src complex promoting CTC formation and metastasis via activating ERK1/2 signal regulatory cascade in BC

We established orthotopic xenograft tumor models to evaluate the therapeutic efficacy of ERK inhibitor SCH772984 (SCH). FOXD1-overexpressing or FOXD1-ctrl MDA231 cells were inoculated into mammary fat pads of NOD/SCID mice (Fig. 6A). After 2 weeks, mice inoculated with FOXD1-overexpressing MDA231 cells were randomly assigned into two groups (FOXD1-SCH, FOXD1-vehicle) that were administered respectively with 25 mg/kg SCH772984 or control vehicle intraperitoneally twice a day (Fig. 6A). Interestingly, we found that CTCs were increased in the FOXD1-vehicle group compared to the control-vehicle group as early as 3 weeks after cell inoculation, indicating that FOXD1 promoted CTC formation from the early stages of BC (Fig. 6B). Notably, a significant reduction of CTC number became detectable in week 3 in the FOXD1-SCH group compared to FOXD1-vehicle (Fig. 6B). Lung metastasis were significantly decreased in the FOXD1-SCH group than FOXD1-vehicle (Fig. 6C). Following the commencement of SCH treatment, we observed an obvious reduction of tumor growth (Fig. 6D) and tumor volume (Fig. 6E) in the FOXD1-SCH group compared to FOXD1-vehicle. Furthermore, IHC confirmed that the expression levels of p-ERK1/2 and Vimentin were decreased in tumor tissues treated with SCH772984 (Fig. 6F).

Collectively, these in vivo data confirm that FOXD1 plays a critical role in promoting CTC formation and metastasis in BC. Blocking FOXD1-dependent ERK1/2 signaling cascade with ERK1/2 inhibitor might serve as an effective therapeutic approach to reduce CTC formation and mitigate breast metastasis.

## Discussion

CTCs play an essential role in tumor metastasis and the underlying molecular mechanisms of CTC formation in BC, particularly in early BC, are unclear. In this study, we sought to identify the regulatory pathway modulating CTC formation in BC. We observed a consistent correlation between the abnormal changes of FOXD1-dependent RalA-ANXA2-Src-ERK1/2 signaling cascade and CTC formation in BC, particularly in early BC. Our data demonstrated that early CTC formation was not a random process; it was under the influence of FOXD1-dependent signaling cascade that BC cells acquired the potential for motility to detach and disseminate from primary tumor foci in BC. RalA, as the upstream activated protein, is required for the binding of Src to ANXA2; RalA forms a complex with ANXA2 and Src, mediates the interaction between Src and ANXA2, and thus promotes the phosphorylation of ANXA2, further inducing the activation of ERK1/2 signal. We also evaluated the clinical benefit of blocking FOXD1’s regulatory cascade in BC, and found that ERK1/2 inhibitor effectively reduced CTC formation and tumor metastasis in BC. This study has profound implications for understanding of CTC formation in early BC patients and provide a novel druggable and effective strategy to reduce early metastasis of BC.

Our study for the first time demonstrated the important role of FOXD1 in promoting CTC formation in BC, particularly in early BC, and provided a new perspective on FOXD1-associated mechanism. Mechanistic studies showed that FOXD1 promoted CTC formation by activating ERK1/2 signaling cascade through RalA-ANXA2-Src complex. It is noteworthy that, besides regulating BC migration and CTC formation, FOXD1 promoted tumor growth as well. This is consistent with some of other previous studies [45, 46]. Our clinical, functional and mechanistic studies consistently supported that FOXD1 primarily modulated CTC formation at the early stage of BC. As tumor was progressing, the effect of FOXD1 on tumor growth became pronounced, which might further quicken the formation of CTCs.

In this study, we first found that FOXD1 directly regulated RalA transcription in BC after using deep sequencing and luciferase assays. RalA expression was regulated by FOXD1 in primary tumors of BC patients, especially in early BC, and positively correlated with the number of CTCs. RalA enhanced CTC formation and metastasis ability of BC cells via the activation of ERK1/2 signal. Furthermore, we found that the activation of ERK1/2 signal by RalA-GTP was dependent on ANXA2 and Src. Src is a non-receptor protein tyrosine kinase. Src is highly overexpressed and activated in different epithelial cancers, especially breast cancer. It is well-documented to promote cancer cell plasticity, motility and invasion, and has been described as a key player in EMT [47, 48]. Tyr23 Phosphorylation of ANXA2 by Src tyrosine kinase promotes proliferation, migration, and invasion of tumor cells [27]. RalA is required for Src-induced phospholipase D (PLD) and MMPs (MMP-2 and 9) activations, thereby promoting tumor formation and cell invasion ability [49, 50]. We first provided strong genetic and biochemical data for RalA-ANXA2-Src interaction and demonstrated that RalA enhanced the interaction between ANXA2 and Src. RalA, ANXA2 and Src formed a complex that executed regulatory functions to increase p-ANXA2 (Tyr23), thereby activating ERK1/2 signaling in BC. Notably, we further observed that RalA-GTP played a critical role in the binding of ANXA2 to Src.

Mitogen-activated protein kinase (MAPK) signaling pathway is involved in signal transduction between cell membrane receptor and nucleus [51]. It often plays an essential regulatory role in the proliferation, apoptosis, and metastasis of malignant tumor cells [52]. As important proteins of MAPK signaling pathway, ERK1/2 can modulate the migration and EMT of tumor cells through regulating ZEB1, SOX2, etc. [53–55]. Our study identified that ERK1/2 signal pathway was a critical downstream effector of RalA and participated in CTC formation and metastasis in BC. ERK1/2 inhibitor significantly reduced CTCs and metastasis from the early stages of BC progression. This confirms the significance of FOXD1-dependent RalA-ANXA2-Src-ERK1/2 signaling cascade in promoting CTC formation and highlights that ERK1/2 inhibitor SCH772984 can be a potential and effective strategy preventing CTC formation and metastasis in BC, particularly in early BC. Moreover, ERK1/2 inhibitor indeed affects some aspects of cellular functions, so clinically, it might be helpful to define optimal targeted delivery of this inhibitor to develop more effective therapeutics for ERK1/2 signal-activated BC patients.

In conclusion, this study reports a FOXD1-dependent RalA-ANXA2-Src complex that promotes CTC formation via activating ERK1/2 signal in BC. The FOXD1 expression in primary BC tissues positively correlates with CTCs in BC patients, particularly in early BC patients. Functional and mechanistic studies provided the first evidence that FOXD1 promoted CTC formation and metastasis in BC by regulating the RalA-ANXA2-Src-ERK1/2 signaling cascade. In vivo experiment with ERK inhibitor validated the effect of FOXD1-dependent cascade on CTC formation and BC metastasis, providing a potential effective approach for preventing BC metastasis (Fig. 6G). Thus, this study not only improves our understanding of molecular mechanisms underlying CTC formation in early BC patients but also provides insight into the therapeutic potential of targeting the FOXD1-dependent ERK1/2 signaling cascade though further understanding this complexity will require a broader view of underlying signal transduction in the future. Tracking or modulating this signaling cascade could offer a viable approach for metastasis risk assessment, prognosis, and prevention of metastasis in early BC patients.

## Conclusions

In summary, our study demonstrates that the FOXD1-dependent RalA-ANXA2-Src complex promotes CTC formation via activating ERK1/2 signal in BC. In vivo treatment with ERK1/2 inhibitor dramatically inhibits the CTC formation and BC metastasis. FOXD1 may serve as a prognostic factor in evaluation of BC metastasis risks, particularly in early-stage BC patients. This signaling cascade is druggable and effective for overcoming CTC formation from the early stages of BC.

## Supplementary Information

**Additional file 1: Supplementary Fig. 1.** (A) RT-qPCR analyses of FOXD1 mRNA in the indicated breast tumor cell lines and MCF-10A. Error bars, SEM. *n* = 3. (B) Western blotting analyses of FOXD1 expression in the indicated breast tumor cell lines and MCF-10A. (C)RT-qPCR analysis of FOXD1 expression in control and sh-FOXD1 cells (MDA231 and MDA468), or control and FOXD1 overexpression cells (MCF-7). Error bars, SEM. *n* = 3. **p < 0.01, ***p<0.001 by Student’s t test. (D) FOXD1 protein expression level in control and FOXD1 knockdown cells (MDA231 and MDA468), or control and FOXD1 overexpression cells (MCF-7) by immunoblotting. **Supplementary Fig. 2.** (A) Cell migration capacity of FOXD1 knockdown BC cells (MDA231 and MDA468), FOXD1 overexpression BC cell (MCF7), and their control cells was determined by the wound healing assays. Scale bar, 1000 μm. Error bars, SD. *n* = 3. **p<0.01 by Student’s t-test. (B) Immunofluorescence images for E-cadherin and Vimentin expression in CtrL and FOXD1 knockdown BC cells (MDA231 and MDA468). Scale bar, 50 μm. (C) The morphology analysis of control and FOXD1 overexpression MCF-7 cells. Scale bar, 100 μm. (D) The cell proliferation in these transfected cells was determined using CCK-8 assays. Error bars, SD. *n* = 3. **p<0.01, ***p<0.001, ****p<0.0001 by Student’s t-test. (E) Expression of FOXD1 across TCGA cancers (with tumor and normal samples). **Supplementary Fig. 3.** (A) Immunoblotting of FOXD1 after MDA231 cells transfected with pEXP-FOXD1 or with control. (B) Immunoblotting of RalA after MDA231 cells transfected with pEXP-RalA or with control. (C) Western blotting assay was used to examine the protein levels of RalA in MCF-7 cell transfected with the control or RalA-siRNAs (si-1, si-2, and si-3). (D) Western blotting assay was used to examine protein levels of FOXD1 in MDA231 cell stably overexpressed the vector or FOXD1. (E) Visualization of genotyping PCR product for each MDA231 single clonal cell line with indicated genotype. (F) Immunoblotting of RalA in MDA231 single clonal cell lines with indicated genotype. (G) WT and RalA KO MDA-MB-231 cells were subjected to transwell migration assays. Scale bar, 250 μm. Error bars, SD. *n* = 3. ***p<0.001 by Student’s t-test. (H) The cell proliferation in MDA231 with WT, RalA-KO#6, or RalA-KO#26 was investigated using CCK-8 assays. Error bars, SD. *n* = 3. ****p<0.0001 by Student’s t-test. ns. = not significant. (I) WT and RalA KO MDA-MB-231 cells were subjected to immunoblotting assays. (J) The cell proliferation in these transfected cells was determined using CCK-8 assays. Error bars, SD. *n* = 3. *p<0.05, **p<0.01, ****p<0.0001 by Student’s t-test. **Supplementary Fig. 4.** (A) FOXD1 overexpression MDA231 in combination with SCH772984 (0, 1.25, 2.5 and 5 μM, 12 h) were subjected to western blotting assays. (B) FOXD1 overexpression MDA231 in combination with SCH772984 (0, 1.25, 2.5 and 5 μM, 12 h) were subjected to transwell migration assays. Scale bar, 250 μm. Error bars, SD. *n* = 3. ***p<0.001 by Student’s t test. (C) Western blotting analyses of E-cadherin, N-cadherin, Vimentin, and GAPDH expression in MDA231 with FOXD1 overexpression alone or in combination with SCH772984 (5 μM, 12 h). (D) MDA231 cells with FOXD1 overexpression were treated with SCH772984 (0, 0.3125, 0.625, 1.25, 2.5 and 5 μM) for 12 hours, and the OD450 was measured by CCK-8 assay. Error bars, SD. *n* = 3. ****p<0.0001 by one-way ANOVA. (E) The predicted model of the RalA-ANXA2-Src ternary interaction. (F) RalA-KO MDA231 cells transfected with constituently inactivated (G26A) or activated (G23V) RalA mutant respectively, were subjected to western blotting assays. For RalA-GTP assays, cells were harvested and subject to RalBP1-RBD pulldown assays to determine RalA-GTP level. Pulldowns were analyzed by immunoblotting with anti-RalA.

**Additional file 2.** Predicted binding sites through JASPAR database, and the sequences of the constructed Luciferase activity reporter assays.

**Additional file 3.** The potential binding partners of RalA identified by immunoprecipitation-LC-MS.

**Additional file 4: Supplementary Table 1.** List of top 5 candidates of RalA-interacting proteins that were identified by co-immunoprecipitation and MS. **Supplementary Table 2.** ZDOCK used to predict the binding sites of RalA and ANXA2. **Supplementary Table 3.** The sequences of the primers. **Supplementary Table 4.** The antibodies applied in this study.

## Data Availability

All data that support the findings of this study are available from the corresponding author upon reasonable request.

## Abbreviations

BC: Breast cancer
CTCs: Circulating tumor cells
AJCC: American Joint Committee on Cancer
EMT: Epithelial-mesenchymal transition
TCGA: The Cancer Genome Atlas
FOXD1: Forkhead Box D1
RalA: RAS Like Proto-Oncogene A
ANXA2: Annexin A2
Erk1/2: Extracellular regulated protein kinases 1/2
DMEM: Dulbecco’s modified Eagle’s medium
shRNA: Short hairpin RNAs
siRNA: Small interfering RNAs
qPCR: Quantitative real time PCR
DAPI: 4,6-diamidino-2-phenylindole
