# Supplementary material for: FOXD1-dependent RalA-ANXA2-Src complex promotes CTC formation in breast cancer
Source: J Exp Clin Cancer Res. 2022 Oct 13;41:301. doi: 10.1186/s13046-022-02504-0 (PMC9558416; doi:10.1186/s13046-022-02504-0)

Supplementary Figure 1

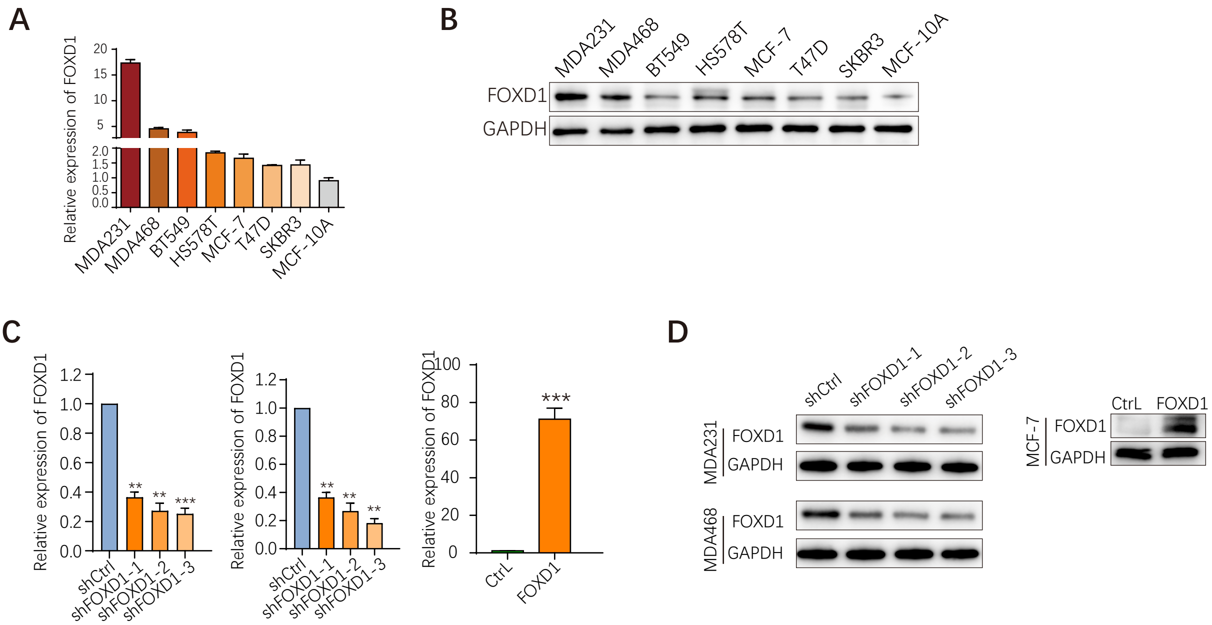

Supplementary Figure 2

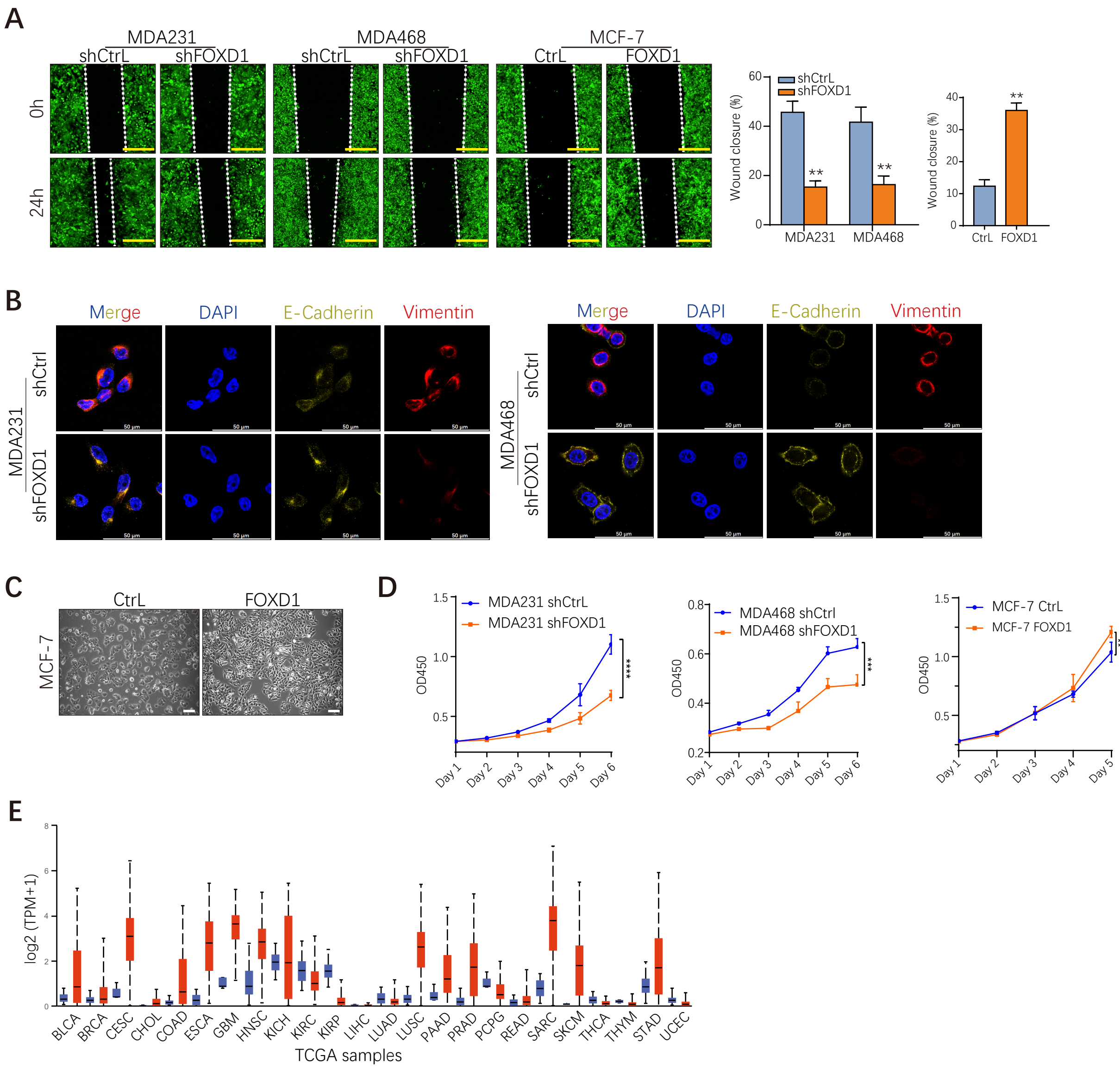

Supplementary Figure 3

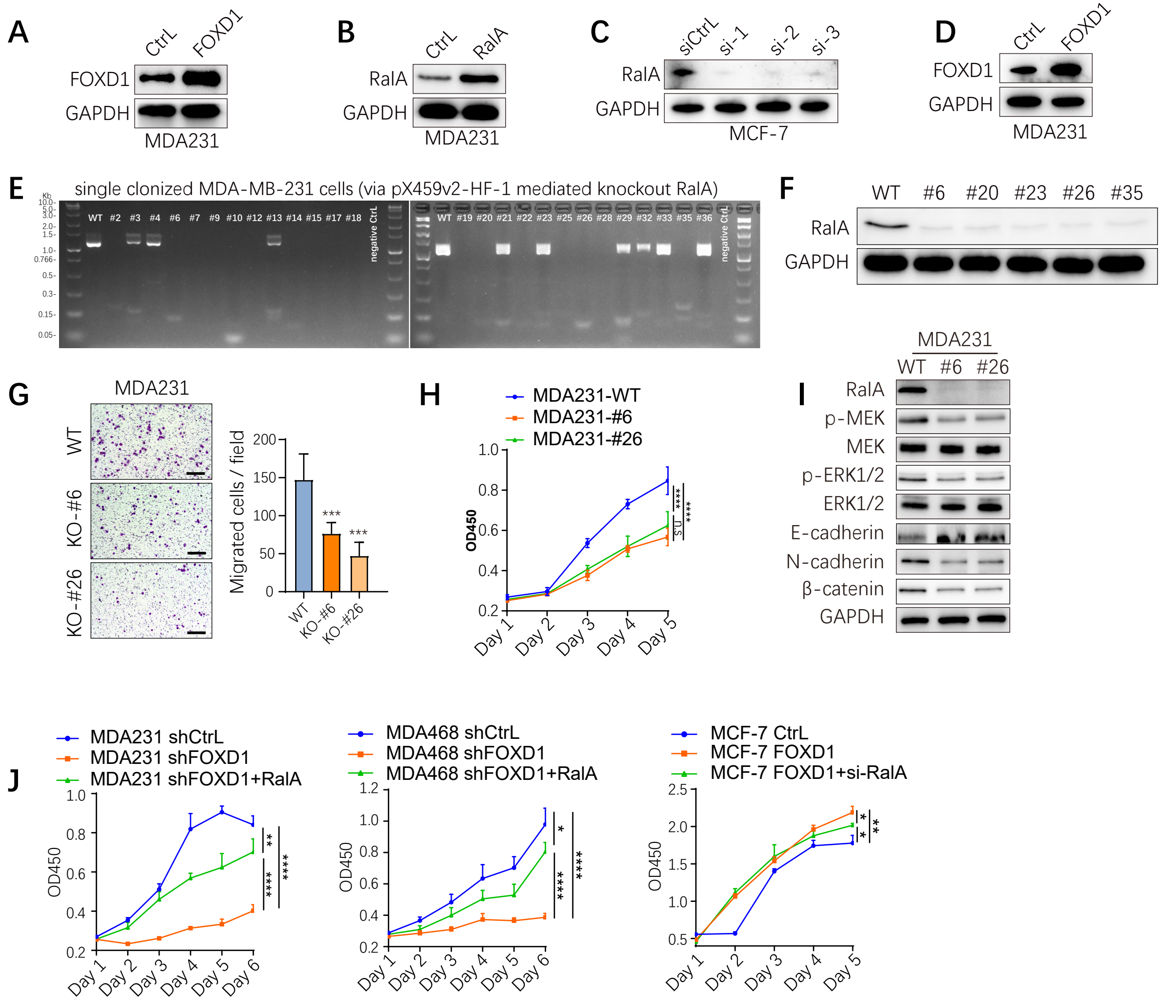

Supplementary Figure 4

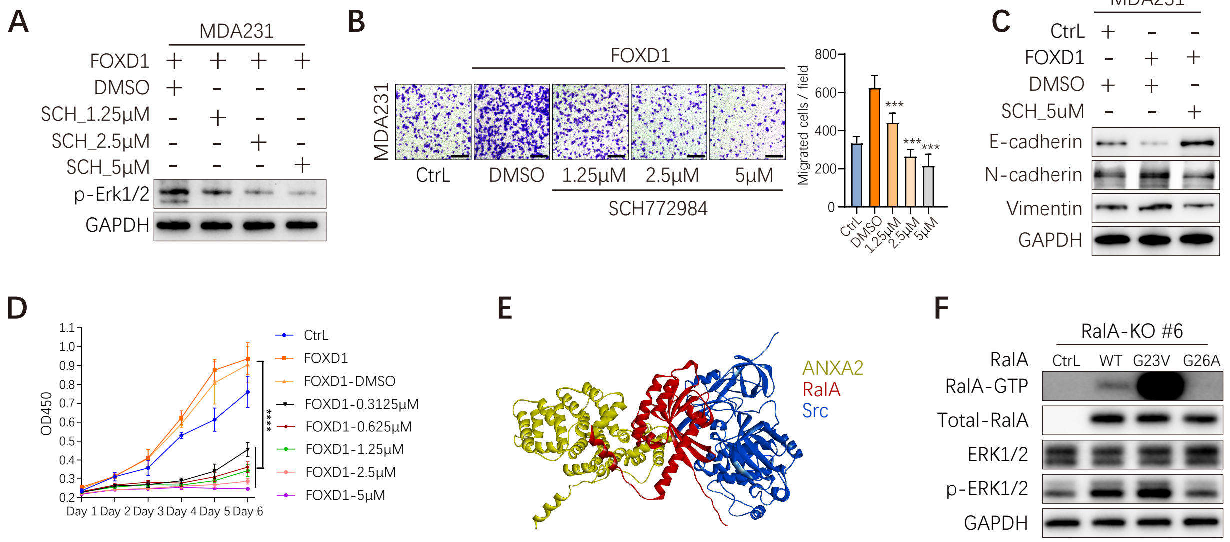

Supplement: Supplementary file 1 — Additional file 1: Supplementary Fig. 1. (A) RT-qPCR analyses of FOXD1 mRNA in the indicated breast tumor cell lines and MCF-10A. Error bars, SEM. n = 3. (B) Western blotting analyses of FOXD1 expression in the indicated breast tumor cell lines and MCF-10A. (C)RT-qPCR analysis of FOXD1 expression in control and sh-FOXD1 cells (MDA231 and MDA468), or control and FOXD1 overexpression cells (MCF-7). Error bars, SEM. n = 3. **p < 0.01, ***p<0.001 by Student’s t test. (D) FOXD1 protein expression level in control and FOXD1 knockdown cells (MDA231 and MDA468), or control and FOXD1 overexpression cells (MCF-7) by immunoblotting. Supplementary Fig. 2. (A) Cell migration capacity of FOXD1 knockdown BC cells (MDA231 and MDA468), FOXD1 overexpression BC cell (MCF7), and their control cells was determined by the wound healing assays. Scale bar, 1000 μm. Error bars, SD. n = 3. **p<0.01 by Student’s t-test. (B) Immunofluorescence images for E-cadherin and Vimentin expression in CtrL and FOXD1 knockdown BC cells (MDA231 and MDA468). Scale bar, 50 μm. (C) The morphology analysis of control and FOXD1 overexpression MCF-7 cells. Scale bar, 100 μm. (D) The cell proliferation in these transfected cells was determined using CCK-8 assays. Error bars, SD. n = 3. **p<0.01, ***p<0.001, ****p<0.0001 by Student’s t-test. (E) Expression of FOXD1 across TCGA cancers (with tumor and normal samples). Supplementary Fig. 3. (A) Immunoblotting of FOXD1 after MDA231 cells transfected with pEXP-FOXD1 or with control. (B) Immunoblotting of RalA after MDA231 cells transfected with pEXP-RalA or with control. (C) Western blotting assay was used to examine the protein levels of RalA in MCF-7 cell transfected with the control or RalA-siRNAs (si-1, si-2, and si-3). (D) Western blotting assay was used to examine protein levels of FOXD1 in MDA231 cell stably overexpressed the vector or FOXD1. (E) Visualization of genotyping PCR product for each MDA231 single clonal cell line with indicated genotype. (F) Immuno [file 13046_2022_2504_MOESM1_ESM.pdf]
