# Supplementary material for: FOXD1-dependent RalA-ANXA2-Src complex promotes CTC formation in breast cancer
Source: J Exp Clin Cancer Res. 2022 Oct 13;41:301. doi: 10.1186/s13046-022-02504-0 (PMC9558416; doi:10.1186/s13046-022-02504-0)
Supplement: Supplementary file 2 — Additional file 2. Predicted binding sites through JASPAR database, and the sequences of the constructed Luciferase activity reporter assays. [file 13046_2022_2504_MOESM2_ESM.pdf]

**Full peak of FOXD1-bingding region on RalA promotor (hg 19, Chr7:39,662,789-39,663,177): Fragment 1+ Fragment 2+ Fragment 3**

GAACATGAGCTGACCCTCTAGAAGCAAGATGGCGGCCTCCGCTCTCCCCTCCTTCCCCCAAGA

ACCCCCTCCTGGTCCCCAAAAGAAAGACAAGGACACCGCGGCAACGACGCCGAGATCCGGG

AACCTCCGCGAGGCCCGCGGCCACTCCCGAGCCCCCGGGAGATGACTTCACCTCGCGGAGC

TCAGAGAGCCGGGGGTGGGGCCGTCGGAAAGACAGCGCGGACAGCCCTCAGCTTGCAGGG

GGCGGAGGCCGGGCAGCCTGCGCCCTCCGAGCCACTGTTCTGCGGCCCAGGCCCATGATCA

CCCTCCTCTCAGCCCACGGACAGGAAGTCGCTCCCCAGCTGCCCCGCCCCGCTCCCCAGCGC

CCCGGAAGTGATCTGTGGCGG

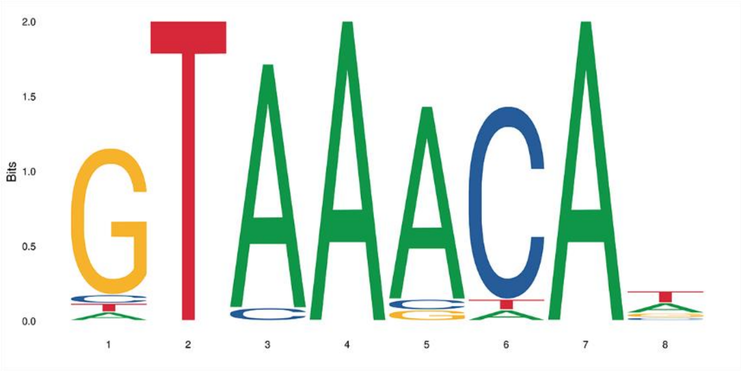


**Fragment 3 WT:**

GAGGCCGGGCAGCCTGCGCCCTCCGAGCCACTGTTCTGCGGCCCAGGCCC**ATGATCAC**CCTC

CTCTCAGCCCACGGACAGGAAGTCGCTCCCCAGCTGCCCCGCCCCGCTCCCCAGCGCCCCGG

AAGTGATCTGTGGCGG

**Fragment 3 Mut:**

GAGGCCGGGCAGCCTGCGCCCTCCGAGCCACTGTTCTGCGGCCCAGGCCC**gaaggtct**CCTCC

TCTCAGCCCACGGACAGGAAGTCGCTCCCCAGCTGCCCCGCCCCGCTCCCCAGCGCCCCGGA

AGTGATCTGTGGCGG

**Additional file 2.** Predicted binding sites through JASPAR database, and the sequences of the constructed Luciferase activity reporter assays.
