# Supplementary material for: FOXD1-dependent RalA-ANXA2-Src complex promotes CTC formation in breast cancer
Source: J Exp Clin Cancer Res. 2022 Oct 13;41:301. doi: 10.1186/s13046-022-02504-0 (PMC9558416; doi:10.1186/s13046-022-02504-0)
Supplement: Supplementary file 3 — Additional file 3. The potential binding partners of RalA identified by immunoprecipitation-LC-MS. [file 13046_2022_2504_MOESM3_ESM.pdf]

Additional file 3. The potential binding partners of RalA identified by immunoprecipitation-LC-MS.

Majority protein IDs	Fasta headers	Number of proteins	Peptides 1	Peptides 2	Unique peptides 1	Unique peptides 2	Mol. weight [kDa]	Sequence coverage 1 [%]	Sequence coverage 2 [%]	LFQ intensity 1	LFQ intensity 2
sp|A2NJV5|KV229_HUMAN;sp|A0A0A0M	>sp|A2NJV5|KV229_HUMAN Immunoglobulin ka	11	2	2	2	2	13.085	16.7	16.7	28100000000	24422000000
sp|P35579|MYH9_HUMAN	>sp|P35579|MYH9_HUMAN Myosin-9 OS=Homo	11	137	118	117	100	226.53	58.9	54.5	44423000000	23608000000
sp|P04264|K2C1_HUMAN;CON__P04264	>sp|P04264|K2C1_HUMAN Keratin, type II cytosk	2	26	38	15	24	66.038	43.2	55.7	7857900000	15126000000
sp|P13645|K1C10_HUMAN;CON__P13645	>sp|P13645|K1C10_HUMAN Keratin, type I cytosk	17	22	29	17	23	58.826	35.3	59.9	8569800000	14969000000
sp|P11233|RALA_HUMAN	>sp|P11233|RALA_HUMAN Ras-related protein Ra	2	0	12	0	12	23.567	0	61.2	0	11317000000
sp|P60709|ACTB_HUMAN	>sp|P60709|ACTB_HUMAN Actin, cytoplasmic 1 (	7	16	21	1	1	41.736	55.5	68.3	8546600000	9072400000
sp|P05787|K2C8_HUMAN;CON__P05787	>sp|P05787|K2C8_HUMAN Keratin, type II cytosk	6	29	32	21	23	53.704	50.3	52.2	6229300000	5848200000
sp|Q15149|PLEC_HUMAN	>sp|Q15149|PLEC_HUMAN Plectin OS=Homo sap	2	124	134	123	133	531.78	29.1	31.4	6015700000	5585100000
sp|P35908|K22E_HUMAN;CON__P35908	>sp|P35908|K22E_HUMAN Keratin, type II cytosk	5	24	32	13	18	65.432	38.3	46.8	2344600000	4392700000
sp|P35527|K1C9_HUMAN;CON__P35527	>sp|P35527|K1C9_HUMAN Keratin, type I cytoske	2	15	22	15	21	62.064	30.7	52.2	1518100000	3479800000
sp|P08727|K1C19_HUMAN;CON__P08727	>sp|P08727|K1C19_HUMAN Keratin, type I cytosk	5	21	27	15	18	44.105	49.2	64.8	3868300000	3408800000
sp|O00410|IPO5_HUMAN	>sp|O00410|IPO5_HUMAN Importin-5 OS=Homo	2	0	36	0	36	123.63	0	45.1	0	3343500000
sp|P05783|K1C18_HUMAN	>sp|P05783|K1C18_HUMAN Keratin, type I cytosk	2	17	19	10	11	48.057	35.1	44.2	3825200000	3219000000
sp|P60660|MYL6_HUMAN	>sp|P60660|MYL6_HUMAN Myosin light polypept	1	7	8	6	6	16.93	48.3	55.6	4977300000	2789900000
sp|P62273|RS29_HUMAN	>sp|P62273|RS29_HUMAN 40S ribosomal protein	1	2	3	2	3	6.6767	32.1	33.9	1613700000	2736100000
sp|Q5VTE0|EF1A3_HUMAN;sp|P68104|EF	>sp|Q5VTE0|EF1A3_HUMAN Putative elongation	3	8	13	8	13	50.184	24.5	39.6	754900000	1804800000
sp|P28799|GRN_HUMAN	>sp|P28799|GRN_HUMAN Progranulin OS=Homo	1	9	10	9	10	63.544	16.7	19.7	1728800000	1599000000
sp|Q9BQE3|TBA1C_HUMAN;sp|Q71U36|T	>sp|Q9BQE3|TBA1C_HUMAN Tubulin alpha-1C (	8	6	15	0	2	49.895	19.8	40.5	355310000	1520500000
sp|P68371|TBB4B_HUMAN;sp|P04350|TBE	>sp|P68371|TBB4B_HUMAN Tubulin beta-4B cha	3	6	17	1	3	49.83	20	47.4	170430000	1254300000
sp|O43790|KRT86_HUMAN;CON__O4379	>sp|O43790|KRT86_HUMAN Keratin, type II cutic	3	1	19	0	2	53.5	1.4	51	0	1202500000
sp|P19105|ML12A_HUMAN;sp|O14950|ML	>sp|P19105|ML12A_HUMAN Myosin regulatory li	3	6	6	6	6	19.794	45.6	45	1429700000	1173200000
sp|Q9NWB6|ARGL1_HUMAN	>sp|Q9NWB6|ARGL1_HUMAN Arginine and glut	1	3	5	3	5	33.216	8.8	14.7	0	371650000
sp|P13647|K2C5_HUMAN;CON__P13647	>sp|P13647|K2C5_HUMAN Keratin, type II cytosk	2	15	25	4	5	62.378	21.9	34.4	358370000	936670000
sp|P67936|TPM4_HUMAN	>sp|P67936|TPM4_HUMAN Tropomyosin alpha-4	3	16	15	9	9	28.521	46.8	50	1502800000	749740000
sp|P23246|SFPQ_HUMAN	>sp|P23246|SFPQ_HUMAN Splicing factor, prolin	1	9	10	8	9	76.149	14.6	17	860620000	713640000
sp|Q14974|IMB1_HUMAN	>sp|Q14974|IMB1_HUMAN Importin subunit beta-	1	0	18	0	18	97.169	0	26.5	0	1000200000
sp|P02533|K1C14_HUMAN;CON__P02533	>sp|P02533|K1C14_HUMAN Keratin, type I cytosk	7	11	17	0	3	51.561	22.9	30.7	165430000	555710000
sp|P05141|ADT2_HUMAN	>sp|P05141|ADT2_HUMAN ADP/ATP translocase	3	4	10	1	4	32.852	13.8	32.9	143550000	518180000
sp|P62269|RS18_HUMAN	>sp|P62269|RS18_HUMAN 40S ribosomal protein	1	5	7	5	7	17.718	23.7	36.8	382230000	493080000
sp|Q59GN2|R39L5_HUMAN;sp|P62891|RL	>sp|Q59GN2|R39L5_HUMAN Putative 60S riboso	2	2	2	2	2	6.3225	23.5	23.5	514340000	478030000
sp|P35580|MYH10_HUMAN	>sp|P35580|MYH10_HUMAN Myosin-10 OS=Hor	2	41	27	25	11	229	21.7	13.6	787620000	417180000
sp|P07437|TBB5_HUMAN;sp|Q9BVA1|TBE	>sp|P07437|TBB5_HUMAN Tubulin beta chain OS	7	7	16	2	2	49.67	22.3	43.7	0	347850000
sp|O95373|IPO7_HUMAN	>sp|O95373|IPO7_HUMAN Importin-7 OS=Homo	2	0	20	0	20	119.52	0	23.7	0	680270000
sp|P02545|LMNA_HUMAN	>sp|P02545|LMNA_HUMAN Prelamin-A/C OS=H	1	14	10	14	10	74.139	26.1	16.9	486560000	341950000
sp|P62861|RS30_HUMAN	>sp|P62861|RS30_HUMAN 40S ribosomal protein	1	2	3	2	3	6.6478	20.3	20.3	344940000	329810000
sp|P62979|RS27A_HUMAN	>sp|P62979|RS27A_HUMAN Ubiquitin-40S riboso	1	2	1	2	1	17.965	20.5	8.3	495760000	327970000
sp|P62851|RS25_HUMAN	>sp|P62851|RS25_HUMAN 40S ribosomal protein	1	4	4	4	4	13.742	24	28	217300000	318760000
sp|B9A064|IGLL5_HUMAN	>sp|B9A064|IGLL5_HUMAN Immunoglobulin lan	1	1	1	1	1	23.063	3.7	3.7	0	188110000
sp|P11021|BIP_HUMAN	>sp|P11021|BIP_HUMAN Endoplasmic reticulum c	1	4	12	4	11	72.332	8.1	23.4	197160000	300540000
sp|P06753|TPM3_HUMAN;sp|P09493|TPM	>sp|P06753|TPM3_HUMAN Tropomyosin alpha-3	2	12	9	5	3	32.95	30.5	30.5	817200000	296130000
sp|P60903|S10AA_HUMAN	>sp|P60903|S10AA_HUMAN Protein S100-A10 O	1	0	2	0	2	11.203	0	27.8	0	51570000
sp|P07355|ANXA2_HUMAN;sp|A6NMY6|A	>sp|P07355|ANXA2_HUMAN Annexin A2 OS=H	2	0	7	0	7	38.604	0	25.7	0	295670000
sp|O75531|BAF_HUMAN	>sp|O75531|BAF_HUMAN Barrier-to-autointegrati	1	3	3	3	3	10.058	29.2	29.2	253230000	291930000
sp|P11142|HSP7C_HUMAN;sp|P54652|HSP	>sp|P11142|HSP7C_HUMAN Heat shock cognate	5	6	14	4	10	70.897	12.2	25.5	201340000	284610000
sp|P51114|FXR1_HUMAN	>sp|P51114|FXR1_HUMAN Fragile X mental retar	2	7	9	5	7	69.72	11.8	15.1	336650000	278840000
sp|P62280|RS11_HUMAN	>sp|P62280|RS11_HUMAN 40S ribosomal protein	1	6	7	6	7	18.431	31	32.3	289560000	277840000
sp|P84098|RL19_HUMAN	>sp|P84098|RL19_HUMAN 60S ribosomal protein	1	1	3	1	3	23.466	8.7	17.3	0	60348000
sp|P09651|ROA1_HUMAN	>sp|P09651|ROA1_HUMAN Heterogeneous nuclea	2	7	9	7	9	38.746	25.8	30.1	257120000	258730000
sp|P68133|ACTS_HUMAN;sp|P68032|ACT	>sp|P68133|ACTS_HUMAN Actin, alpha skeletal n	4	9	12	1	1	42.051	22.5	27.6	276370000	251030000
sp|Q9Y383|LC7L2_HUMAN;sp|Q9NQ29|LU	>sp|Q9Y383|LC7L2_HUMAN Putative RNA-bindi	2	8	7	8	7	46.513	23	19.9	298410000	234920000
sp|Q15233|NONO_HUMAN	>sp|Q15233|NONO_HUMAN Non-POU domain-c	1	5	6	4	5	54.231	11.3	14	331210000	232890000
sp|P11940|PABP1_HUMAN	>sp|P11940|PABP1_HUMAN Polyadenylate-bindin	4	8	11	5	8	70.67	14.9	14.5	289690000	220780000
sp|P22626|ROA2_HUMAN	>sp|P22626|ROA2_HUMAN Heterogeneous nuclea	1	6	7	6	7	37.429	20.7	24.1	259770000	204550000
sp|P46782|RS5_HUMAN	>sp|P46782|RS5_HUMAN 40S ribosomal protein S	1	2	6	2	6	22.876	12.3	28.9	97066000	200770000
sp|P62701|RS4X_HUMAN;sp|P22090|RS4Y	>sp|P62701|RS4X_HUMAN 40S ribosomal protein	3	7	8	7	8	29.597	19.4	26.2	174500000	199490000
sp|P05976|MYL1_HUMAN;sp|P08590|MYL	>sp|P05976|MYL1_HUMAN Myosin light chain 1/	2	1	1	1	1	21.145	8.2	8.2	316730000	196440000
sp|P47914|RL29_HUMAN	>sp|P47914|RL29_HUMAN 60S ribosomal protein	1	0	1	0	1	17.752	0	9.4	0	37369000
sp|P48668|K2C6C_HUMAN;CON__P4866	>sp|P48668|K2C6C_HUMAN Keratin, type II cytos	3	12	25	0	0	60.024	16	34.8	0	275150000
sp|P15880|RS2_HUMAN	>sp|P15880|RS2_HUMAN 40S ribosomal protein S	1	2	9	2	9	31.324	6.5	27.3	41426000	181370000
sp|Q6WCQ1|MPRIP_HUMAN	>sp|Q6WCQ1|MPRIP_HUMAN Myosin phosphata	2	12	6	12	6	116.53	16.4	8.9	369400000	170920000
sp|P08708|RS17_HUMAN	>sp|P08708|RS17_HUMAN 40S ribosomal protein	1	3	2	3	2	15.55	23.7	16.3	209190000	163260000
sp|P60866|RS20_HUMAN	>sp|P60866|RS20_HUMAN 40S ribosomal protein	1	3	3	3	3	13.373	28.6	28.6	127990000	158850000
sp|P23396|RS3_HUMAN	>sp|P23396|RS3_HUMAN 40S ribosomal protein S	1	4	6	4	6	26.688	16	25.9	77430000	158670000
sp|P26373|RL13_HUMAN	>sp|P26373|RL13_HUMAN 60S ribosomal protein	1	6	3	6	3	24.261	22.7	13.7	202910000	154280000
sp|P62913|RL11_HUMAN	>sp|P62913|RL11_HUMAN 60S ribosomal protein	1	1	3	1	3	20.252	7.9	17.4	0	89344000

sp|O76021|RL1D1 HUMAN	>sp|O76021|RL1D1 HUMAN Ribosomal L1 doma	1	0	1	0	1	54.972	0	2.4	0	194710000
sp|P62241|RS8 HUMAN	>sp|P62241|RS8 HUMAN 40S ribosomal protein S	1	4	2	4	2	24.205	21.2	11.5	0	79275000
sp|Q04941|PLP2 HUMAN	>sp|Q04941|PLP2 HUMAN Proteolipid protein 2 C	1	0	1	0	1	16.691	0	8.6	0	43487000
sp|P42166|LAP2A HUMAN	>sp|P42166|LAP2A HUMAN Lamina-associated p	2	9	4	9	4	75.491	19.2	7.5	161830000	142040000
sp|P18085|ARF4 HUMAN	>sp|P18085|ARF4 HUMAN ADP-ribosylation fact	1	0	3	0	3	20.511	0	15	0	94225000
sp|P42766|RL35_HUMAN	>sp|P42766|RL35_HUMAN 60S ribosomal protein	1	3	2	3	2	14.551	18.7	13.8	130950000	135530000
sp|P61586|RHOA_HUMAN;sp|P08134|RHC	>sp|P61586|RHOA_HUMAN Transforming protein	2	0	1	0	1	21.768	0	5.7	0	67828000
sp|P08670|VIME HUMAN	>sp|P08670|VIME HUMAN Vimentin OS=Homo s	3	10	5	7	3	53.651	18	9.2	142930000	128930000
sp|P62266|RS23 HUMAN	>sp|P62266|RS23 HUMAN 40S ribosomal protein	1	6	4	6	4	15.807	30.1	22.4	132290000	128430000
sp|P62249|RS16 HUMAN	>sp|P62249|RS16 HUMAN 40S ribosomal protein	1	3	6	3	6	16.445	17.1	43.2	116360000	126140000
sp|Q99878|H2A1J HUMAN;sp|Q96KK5|H2	>sp|Q99878|H2A1J HUMAN Histone H2A type 1-	15	2	3	2	3	13.936	12.5	27.3	160090000	125510000
sp|P26038|MOES HUMAN	>sp|P26038|MOES HUMAN Moesin OS=Homo sa	1	4	7	1	4	67.819	5.7	10.1	78179000	122900000
sp|P04406|G3P HUMAN	>sp|P04406|G3P HUMAN Glyceraldehyde-3-phosp	2	0	8	0	8	36.053	0	30.7	0	141560000
sp|P84090|ERH_HUMAN	>sp|P84090|ERH_HUMAN Enhancer of rudimentar	1	1	1	1	1	12.259	10.6	10.6	0	41077000
sp|Q04837|SSBP_HUMAN	>sp|Q04837|SSBP_HUMAN Single-stranded DNA-	1	3	3	3	3	17.259	21.6	21.6	134480000	106580000
sp|P46783|RS10 HUMAN;sp|Q9NQ39|RS1(	>sp|P46783|RS10 HUMAN 40S ribosomal protein	2	2	4	2	4	18.898	14.5	27.9	98984000	104060000
sp|P39019|RS19 HUMAN	>sp|P39019|RS19 HUMAN 40S ribosomal protein	1	2	3	2	3	16.06	6.9	13.1	72613000	102490000
sp|P62750|RL23A HUMAN	>sp|P62750|RL23A HUMAN 60S ribosomal protei	1	4	3	4	3	17.695	26.9	14.7	111370000	99986000
sp|P35268|RL22 HUMAN	>sp|P35268|RL22 HUMAN 60S ribosomal protein	1	0	2	0	2	14.787	0	18.8	0	32192000
sp|P63173|RL38 HUMAN	>sp|P63173|RL38 HUMAN 60S ribosomal protein	1	4	2	4	2	8.2178	50	35.7	210480000	96778000
sp|P46779|RL28 HUMAN	>sp|P46779|RL28 HUMAN 60S ribosomal protein	1	2	3	2	3	15.747	15.3	13.9	0	62780000
sp|Q8WWM7|ATX2L_HUMAN	>sp|Q8WWM7|ATX2L_HUMAN Ataxin-2-like prc	1	3	9	3	9	113.37	4	9.7	97515000	94315000
sp|Q9BYT5|KRA22_HUMAN;sp|Q9BYU5|l	>sp|Q9BYT5|KRA22_HUMAN Keratin-associated	5	0	1	0	1	12.957	0	8.1	0	52198000
sp|Q14498|RBM39 HUMAN	>sp|Q14498|RBM39 HUMAN RNA-binding protei	2	4	7	4	7	59.379	9.6	15.1	71566000	92643000
sp|P31943|HNRH1 HUMAN;sp|P55795|HN	>sp|P31943|HNRH1 HUMAN Heterogeneous nucl	3	3	3	3	3	49.229	9.6	9.6	75763000	91122000
sp|Q02878|RL6 HUMAN	>sp|Q02878|RL6 HUMAN 60S ribosomal protein I	1	2	4	2	4	32.728	7.6	16.3	89137000	90591000
sp|Q96P70|IPO9 HUMAN	>sp|Q96P70|IPO9 HUMAN Importin-9 OS=Homo	1	0	12	0	12	115.96	0	13.7	0	304500000
sp|Q14764|MVP HUMAN	>sp|Q14764|MVP HUMAN Major vault protein OS	1	11	4	11	4	99.326	12.5	4.5	162640000	89013000
sp|P46776|RL27A HUMAN	>sp|P46776|RL27A HUMAN 60S ribosomal protei	1	3	2	3	2	16.561	20.9	15.5	83721000	84703000
sp|P40939|ECHA_HUMAN	>sp|P40939|ECHA_HUMAN Trifunctional enzyme	1	0	12	0	12	82.999	0	19	0	293890000
sp|P61353|RL27_HUMAN	>sp|P61353|RL27_HUMAN 60S ribosomal protein	1	0	2	0	2	15.798	0	12.5	0	41503000
sp|P39023|RL3 HUMAN	>sp|P39023|RL3 HUMAN 60S ribosomal protein L	1	3	2	3	2	46.108	7.2	5.2	79128000	81759000
sp|P17844|DDX5 HUMAN	>sp|P17844|DDX5 HUMAN Probable ATP-depenc	1	2	6	1	3	69.147	3.7	10.6	81272000	80754000
sp|P62277|RS13 HUMAN	>sp|P62277|RS13 HUMAN 40S ribosomal protein	1	1	2	1	2	17.222	4.6	11.3	0	61092000
sp|Q9NRW1|RAB6B HUMAN;sp|P20340|R	>sp|Q9NRW1|RAB6B HUMAN Ras-related protei	3	0	1	0	1	23.461	0	5.3	0	83328000
sp|P63000|RAC1 HUMAN;sp|P60763|RAC:	>sp|P63000|RAC1 HUMAN Ras-related C3 botulir	3	0	2	0	2	21.45	0	9.4	0	50102000
sp|Q00325|MPCP HUMAN	>sp|Q00325|MPCP HUMAN Phosphate carrier pro	1	1	1	1	1	40.094	3.3	3.3	0	111950000
sp|Q06830|PRDX1_HUMAN	>sp|Q06830|PRDX1_HUMAN Peroxiredoxin-1 OS	2	2	4	2	4	22.11	9.5	25.6	45228000	75978000
sp|Q6NXT2|H3C_HUMAN;sp|Q71DI3|H32_	>sp|Q6NXT2|H3C_HUMAN Histone H3.3C OS=H	5	2	2	2	2	15.214	11.9	11.9	74573000	75376000
sp|P01859|IGHG2 HUMAN	>sp|P01859|IGHG2 HUMAN Immunoglobulin hea	1	1	1	1	1	35.9	2.8	2.8	0	80343000
sp|Q9UN86|G3BP2 HUMAN	>sp|Q9UN86|G3BP2 HUMAN Ras GTPase-activat	1	1	3	1	3	54.12	2.7	11	95321000	71400000
sp|P18621|RL17 HUMAN	>sp|P18621|RL17 HUMAN 60S ribosomal protein	1	1	2	1	2	21.397	7.6	13	0	54492000
sp|P49207|RL34 HUMAN	>sp|P49207|RL34 HUMAN 60S ribosomal protein	1	2	3	2	3	13.293	12.8	20.5	72643000	65726000
sp|P62826|RAN HUMAN	>sp|P62826|RAN HUMAN GTP-binding nuclear p	1	0	3	0	3	24.423	0	14.4	0	65420000
sp|Q86V81|THOC4 HUMAN	>sp|Q86V81|THOC4 HUMAN THO complex subu	1	1	1	1	1	26.888	4.3	4.3	65326000	65420000
sp|P35637|FUS_HUMAN	>sp|P35637|FUS_HUMAN RNA-binding protein F	1	4	3	2	2	53.425	6.3	6.3	51079000	64605000
sp|P62899|RL31_HUMAN	>sp|P62899|RL31_HUMAN 60S ribosomal protein	1	1	2	1	2	14.463	7.2	18.4	0	41349000
sp|P61247|RS3A_HUMAN	>sp|P61247|RS3A_HUMAN 40S ribosomal protein	1	1	2	1	2	29.945	4.5	6.4	0	96501000
sp|P62910|RL32 HUMAN	>sp|P62910|RL32 HUMAN 60S ribosomal protein	1	1	1	1	1	15.86	9.6	9.6	59536000	62611000
sp|Q07020|RL18 HUMAN	>sp|Q07020|RL18 HUMAN 60S ribosomal protein	1	0	2	0	2	21.634	0	11.7	0	38738000
sp|P08779|K1C16 HUMAN;CON P08779	>sp|P08779|K1C16 HUMAN Keratin, type I cytosk	2	8	15	0	6	51.267	15.9	31.3	0	153730000
sp|P40429|RL13A HUMAN;sp|Q6NVV1|R1	>sp|P40429|RL13A HUMAN 60S ribosomal protei	2	2	2	2	2	23.577	9.4	9.4	55333000	61087000
sp|P60953|CDC42 HUMAN	>sp|P60953|CDC42 HUMAN Cell division control	1	0	2	0	2	21.258	0	11	0	46714000
sp|P51991|ROA3 HUMAN	>sp|P51991|ROA3 HUMAN Heterogeneous nuclea	1	2	1	2	1	39.594	9.3	3.4	70730000	60331000
sp|P61254|RL26_HUMAN;sp|Q9UNX3|RL2	>sp|P61254|RL26_HUMAN 60S ribosomal protein	2	3	4	3	4	17.258	18.6	17.2	48077000	57014000
sp|Q9Y2W1|TR150_HUMAN	>sp|Q9Y2W1|TR150_HUMAN Thyroid hormone re	1	3	4	3	4	108.66	4.1	4.3	78348000	56990000
sp|P62888|RL30 HUMAN	>sp|P62888|RL30 HUMAN 60S ribosomal protein	1	0	3	0	3	12.784	0	37.4	0	30853000
sp|Q9P0K7|RAI14 HUMAN	>sp|Q9P0K7|RAI14 HUMAN Ankycorbin OS=Hoı	1	5	3	5	3	110.04	4.9	3.2	43383000	55063000
sp|Q15323|K1H1 HUMAN;CON Q9UE12	>sp|Q15323|K1H1 HUMAN Keratin, type I cuticul	3	1	12	0	2	47.237	1.7	28.8	0	130020000
sp|Q02543|RL18A HUMAN	>sp|Q02543|RL18A HUMAN 60S ribosomal prote	1	1	2	1	2	20.762	5.7	13.1	52307000	54387000
sp|Q13283|G3BP1 HUMAN	>sp|Q13283|G3BP1 HUMAN Ras GTPase-activati	1	2	2	2	2	52.164	6.9	4.1	63846000	54265000
sp|P61978|HNRPK HUMAN	>sp|P61978|HNRPK HUMAN Heterogeneous nucl	1	4	5	4	5	50.976	10.8	13	48257000	52761000
sp|P62424|RL7A_HUMAN	>sp|P62424|RL7A_HUMAN 60S ribosomal protein	1	2	2	2	2	29.995	7.5	8.3	0	47918000
sp|P08238|HS90B_HUMAN	>sp|P08238|HS90B_HUMAN Heat shock protein H	3	2	7	1	3	83.263	2.6	9.4	0	154180000
sp|P04792|HSPB1 HUMAN	>sp|P04792|HSPB1 HUMAN Heat shock protein b	1	0	3	0	3	22.782	0	18	0	61870000
sp|P27635|RL10 HUMAN;sp|Q96L21|RL10	>sp|P27635|RL10 HUMAN 60S ribosomal protein	2	2	2	2	2	24.604	7.9	7.9	0	34895000
sp|P30050|RL12 HUMAN	>sp|P30050|RL12 HUMAN 60S ribosomal protein	1	2	2	2	2	17.818	18.8	18.8	129130000	49306000
sp|Q8N9Q2|SR1IP HUMAN	>sp|Q8N9Q2|SR1IP HUMAN Protein SREK1IP1 (	1	1	1	1	1	18.177	7.7	7.7	0	30431000
sp|P61026|RAB10 HUMAN;sp|P51153|RAI	>sp|P61026|RAB10 HUMAN Ras-related protein R	5	0	2	0	1	22.541	0	11.5	0	55589000

sp|Q5T750|XP32_HUMAN	>sp|Q5T750|XP32_HUMAN Skin-specific protein	1	0	2	0	2	26.238	0	3.6	0	45200000
sp|P68363|TBA1B_HUMAN;sp|P68366|TB	>sp|P68363|TBA1B_HUMAN Tubulin alpha-1B ch	2	7	14	1	1	50.151	22.6	36.6	0	82752000
sp|P17066|HSP76_HUMAN;sp|P48741|HSP	>sp|P17066|HSP76_HUMAN Heat shock 70 kDa p	2	3	4	1	1	71.027	5.1	6.2	0	145590000
sp|P62244|RS15A_HUMAN	>sp|P62244|RS15A_HUMAN 40S ribosomal protei	1	0	2	0	2	14.839	0	13.1	0	30951000
sp|Q07021|C1QBP_HUMAN	>sp|Q07021|C1QBP_HUMAN Complement compo	1	0	2	0	2	31.362	0	9.6	0	49829000
sp|Q03135|CAV1_HUMAN;sp|P56539|CAV	>sp|Q03135|CAV1_HUMAN Caveolin-1 OS=Hom	2	0	2	0	2	20.471	0	10.1	0	35962000
sp|Q14525|KT33B_HUMAN;CON__Q1452	>sp|Q14525|KT33B_HUMAN Keratin, type I cutic	4	1	11	0	3	46.213	1.7	29	0	96960000
sp|P23528|COF1_HUMAN	>sp|P23528|COF1_HUMAN Cofilin-1 OS=Homo s	1	0	2	0	2	18.502	0	15.7	0	35420000
sp|Q9NZ01|TECR_HUMAN	>sp|Q9NZ01|TECR_HUMAN Very-long-chain eno	1	0	2	0	2	36.034	0	6.5	0	43787000
sp|P09382|LEG1_HUMAN	>sp|P09382|LEG1_HUMAN Galectin-1 OS=Homo	1	1	2	1	2	14.716	11.1	24.4	0	29296000
sp|P42677|RS27_HUMAN;sp|Q71UM5|RS2	>sp|P42677|RS27_HUMAN 40S ribosomal protein	2	1	1	1	1	9.461	9.5	15.5	0	12964000
sp|O00159|MYO1C_HUMAN	>sp|O00159|MYO1C_HUMAN Unconventional my	1	2	3	2	3	121.68	1.8	2.6	41743000	39629000
sp|P05386|RLA1_HUMAN	>sp|P05386|RLA1_HUMAN 60S acidic ribosomal	1	1	1	1	1	11.514	14	14	0	9327000
sp|P31949|S10AB_HUMAN	>sp|P31949|S10AB_HUMAN Protein S100-A11 O	1	0	2	0	2	11.74	0	20	0	15370000
sp|Q96KP1|EXOC2_HUMAN	>sp|Q96KP1|EXOC2_HUMAN Exocyst complex c	1	0	9	0	9	104.07	0	11.1	0	145740000
sp|P55084|ECHB_HUMAN	>sp|P55084|ECHB_HUMAN Trifunctional enzyme	1	0	3	0	3	51.294	0	5.9	0	73579000
sp|P50914|RL14_HUMAN	>sp|P50914|RL14_HUMAN 60S ribosomal protein	1	1	1	1	1	23.432	5.6	5.6	0	16888000
sp|Q92973|TNPO1_HUMAN;sp|O14787|TN	>sp|Q92973|TNPO1_HUMAN Transportin-1 OS=	2	0	4	0	4	102.35	0	6.1	0	114750000
sp|P61313|RL15_HUMAN	>sp|P61313|RL15_HUMAN 60S ribosomal protein	1	0	3	0	3	24.146	0	14.7	0	29901000
sp|P32969|RL9_HUMAN	>sp|P32969|RL9_HUMAN 60S ribosomal protein L	1	0	1	0	1	21.863	0	3.6	0	23485000
sp|P53999|TCP4_HUMAN	>sp|P53999|TCP4_HUMAN Activated RNA polym	1	1	1	1	1	14.395	10.2	10.2	0	12826000
sp|P36542|ATPG_HUMAN	>sp|P36542|ATPG_HUMAN ATP synthase subunit	1	0	3	0	3	32.996	0	9.7	0	32510000
sp|P14618|KPYM_HUMAN	>sp|P14618|KPYM_HUMAN Pyruvate kinase PK	1	1	4	1	4	57.936	3	11.9	0	78005000
sp|O15427|MOT4_HUMAN	>sp|O15427|MOT4_HUMAN Monocarboxylate tra	1	0	2	0	2	49.469	0	5.6	0	40197000
sp|Q07955|SRSF1_HUMAN	>sp|Q07955|SRSF1_HUMAN Serine/arginine-rich	1	1	2	1	2	27.744	4.8	8.9	0	28152000
sp|P04899|GNAI2_HUMAN;sp|P63096|GN	>sp|P04899|GNAI2_HUMAN Guanine nucleotide-	10	0	3	0	3	40.45	0	10.4	0	36734000
sp|Q8NHW5|RLA0L_HUMAN;sp|P05388|R	>sp|Q8NHW5|RLA0L_HUMAN 60S acidic riboso	2	0	1	0	1	34.364	0	3.5	0	29652000
sp|O00299|CLIC1_HUMAN	>sp|O00299|CLIC1_HUMAN Chloride intracellula	1	0	2	0	2	26.922	0	8.7	0	31298000
sp|P0DP25|CALM3_HUMAN;sp|P0DP24|C	>sp|P0DP25|CALM3_HUMAN Calmodulin-3 OS=	3	0	1	0	1	16.837	0	11.4	0	18595000
sp|P78386|KRT85_HUMAN;CON__P78386	>sp|P78386|KRT85_HUMAN Keratin, type II cutic	2	1	11	0	2	55.802	1.4	23.1	0	63379000
sp|Q9UM00|TMCO1_HUMAN	>sp|Q9UM00|TMCO1_HUMAN Calcium load-acti	1	0	2	0	2	27.079	0	9.6	0	15261000
sp|P62879|GBB2_HUMAN;sp|Q9HAV0|GB	>sp|P62879|GBB2_HUMAN Guanine nucleotide-b	4	1	2	1	2	37.331	2.9	6.8	0	23451000
sp|P12236|ADT3_HUMAN	>sp|P12236|ADT3_HUMAN ADP/ATP translocase	1	3	9	0	3	32.866	10.4	28.5	0	35112000
sp|P56134|ATPK_HUMAN	>sp|P56134|ATPK_HUMAN ATP synthase subunit	1	0	2	0	2	10.918	0	25.5	0	6803400
sp|P83731|RL24_HUMAN	>sp|P83731|RL24_HUMAN 60S ribosomal protein	1	1	1	1	1	17.779	5.1	5.1	0	13384000
sp|P60468|SC61B_HUMAN	>sp|P60468|SC61B_HUMAN Protein transport prot	1	0	1	0	1	9.9743	0	15.6	0	9967500
sp|P61769|B2MG_HUMAN	>sp|P61769|B2MG_HUMAN Beta-2-microglobulin	1	0	1	0	1	13.714	0	8.4	0	9519100
sp|P21796|VDAC1_HUMAN	>sp|P21796|VDAC1_HUMAN Voltage-dependent	1	0	1	0	1	30.772	0	3.9	0	26852000
sp|P25705|ATPA_HUMAN	>sp|P25705|ATPA_HUMAN ATP synthase subunit	1	0	3	0	3	59.75	0	6.5	0	48797000
sp|Q96AG4|LRC59_HUMAN	>sp|Q96AG4|LRC59_HUMAN Leucine-rich repeat	1	0	3	0	3	34.93	0	12.1	0	21222000
sp|Q8TEX9|IPO4_HUMAN	>sp|Q8TEX9|IPO4_HUMAN Importin-4 OS=Hom	1	0	8	0	8	118.71	0	9.7	0	78552000
sp|P07900|HS90A_HUMAN	>sp|P07900|HS90A_HUMAN Heat shock protein H	3	2	7	1	3	84.659	2.7	10.7	0	47676000
sp|Q15365|PCBP1_HUMAN;sp|Q15366|PC	>sp|Q15365|PCBP1_HUMAN Poly(rC)-binding pr	3	0	1	0	1	37.497	0	3.1	0	23393000
sp|P84103|SRSF3_HUMAN	>sp|P84103|SRSF3_HUMAN Serine/arginine-rich s	1	0	1	0	1	19.329	0	12.8	0	9585000
sp|P62753|RS6_HUMAN	>sp|P62753|RS6_HUMAN 40S ribosomal protein S	1	1	2	1	2	28.68	4.4	4.8	0	9839400
sp|Q14103|HNRPD_HUMAN	>sp|Q14103|HNRPD_HUMAN Heterogeneous nuc	1	0	1	0	1	38.434	0	3.9	0	15965000
sp|P16070|CD44_HUMAN	>sp|P16070|CD44_HUMAN CD44 antigen OS=Ho	1	0	2	0	2	81.537	0	3	0	32733000
sp|Q6NZI2|CAVN1_HUMAN	>sp|Q6NZI2|CAVN1_HUMAN Caveolae-associate	1	0	1	0	1	43.476	0	2.8	0	18770000
sp|P27105|STOM_HUMAN	>sp|P27105|STOM_HUMAN Erythrocyte band 7 in	1	0	1	0	1	31.73	0	2.4	0	17291000
sp|P04439|HLAA_HUMAN;sp|P30511|HLA	>sp|P04439|HLAA_HUMAN HLA class I histocom	4	0	2	0	2	40.84	0	4.9	0	23186000
sp|Q13310|PABP4_HUMAN	>sp|Q13310|PABP4_HUMAN Polyadenylate-bindi	3	6	6	3	3	70.782	11.6	8.7	0	35035000
sp|Q14739|LBR_HUMAN	>sp|Q14739|LBR_HUMAN Delta(14)-sterol reduct	1	0	2	0	2	70.702	0	2.9	0	22486000
sp|P27658|CO8A1_HUMAN	>sp|P27658|CO8A1_HUMAN Collagen alpha-1(VI	1	2	2	2	2	73.363	3.9	3.9	24193000	19804000
sp|P14649|MYL6B_HUMAN	>sp|P14649|MYL6B_HUMAN Myosin light chain	1	1	3	0	1	22.764	6.2	15.9	0	14981000
sp|P51148|RAB5C_HUMAN	>sp|P51148|RAB5C_HUMAN Ras-related protein	1	0	1	0	1	23.482	0	5.6	0	11699000
sp|Q02978|M2OM_HUMAN	>sp|Q02978|M2OM_HUMAN Mitochondrial 2-oxo	1	0	1	0	1	34.061	0	5.1	0	17721000
sp|P45880|VDAC2_HUMAN	>sp|P45880|VDAC2_HUMAN Voltage-dependent	1	0	1	0	1	31.566	0	2.7	0	16525000
sp|P49411|EFTU_HUMAN	>sp|P49411|EFTU_HUMAN Elongation factor Tu,	1	0	1	0	1	49.541	0	1.5	0	30745000
sp|Q13501|SQSTM_HUMAN	>sp|Q13501|SQSTM_HUMAN Sequestosome-1 O	1	0	1	0	1	47.687	0	3.2	0	20257000
sp|P15311|EZRI_HUMAN;sp|P35241|RADI	>sp|P15311|EZRI_HUMAN Ezrin OS=Homo sapie	2	5	5	2	2	69.412	7.7	7	0	31752000
sp|P63104|1433Z_HUMAN;sp|P27348|1433	>sp|P63104|1433Z_HUMAN 14-3-3 protein zeta/de	7	0	2	0	2	27.745	0	9.8	0	13735000
sp|P08865|RSSA_HUMAN	>sp|P08865|RSSA_HUMAN 40S ribosomal protein	1	0	1	0	1	32.854	0	4.4	0	12381000
sp|P55060|XPO2_HUMAN	>sp|P55060|XPO2_HUMAN Exportin-2 OS=Homo	1	0	4	0	4	110.42	0	4.4	0	48808000
sp|P02538|K2C6A_HUMAN;CON__P0253	>sp|P02538|K2C6A_HUMAN Keratin, type II cytos	3	12	25	0	1	60.044	16	34.8	0	27911000
sp|P22061|PIMT_HUMAN	>sp|P22061|PIMT_HUMAN Protein-L-isoaspartate	1	1	1	1	1	24.636	6.2	6.2	0	10892000
sp|P00338|LDHA_HUMAN	>sp|P00338|LDHA_HUMAN L-lactate dehydrogen	1	0	1	0	1	36.688	0	3	0	15866000
sp|Q8NC51|PAIRB_HUMAN	>sp|Q8NC51|PAIRB_HUMAN Plasminogen activa	1	0	2	0	2	44.965	0	6.4	0	15705000
sp|P07910|HNRPC_HUMAN	>sp|P07910|HNRPC_HUMAN Heterogeneous nucl	1	0	2	0	2	33.67	0	7.2	0	14784000
sp|Q5T749|KPRP_HUMAN	>sp|Q5T749|KPRP_HUMAN Keratinocyte proline-	1	2	2	2	2	64.135	3.6	3.6	0	21033000

sp|P57088|TMM33_HUMAN	>sp|P57088|TMM33_HUMAN Transmembrane pro	1	0	1	0	1	27.978	0	4	0	8965700
sp|P52272|HNRPM_HUMAN	>sp|P52272|HNRPM_HUMAN Heterogeneous nuc	1	1	4	1	4	77.515	2.2	7.8	0	38230000
sp|Q9UBM7|DHCR7_HUMAN	>sp|Q9UBM7|DHCR7_HUMAN 7-dehydrocholeste	1	0	1	0	1	54.489	0	2.7	0	9546000
sp|P50402|EMD_HUMAN	>sp|P50402|EMD_HUMAN Emerin OS=Homo sap	1	0	1	0	1	28.994	0	4.7	0	8984400
sp|P12268|IMDH2_HUMAN	>sp|P12268|IMDH2_HUMAN Inosine-5-monophos	1	1	3	1	3	55.804	1.6	6	0	16268000
sp|Q16891|MIC60_HUMAN	>sp|Q16891|MIC60_HUMAN MICOS complex sub	1	0	3	0	3	83.677	0	4.5	0	32488000
sp|P13010|XRCC5_HUMAN	>sp|P13010|XRCC5_HUMAN X-ray repair cross-co	1	1	2	1	2	82.704	1.1	2.6	0	26706000
sp|P29692|EF1D_HUMAN	>sp|P29692|EF1D_HUMAN Elongation factor 1-de	1	0	1	0	1	31.121	0	3.2	0	10447000
sp|Q9NVI7|ATD3A_HUMAN;sp|Q5T9A4|A	>sp|Q9NVI7|ATD3A_HUMAN ATPase family AA	2	0	1	0	1	71.368	0	1.7	0	21310000
sp|Q9H0U4|RAB1B_HUMAN;sp|Q92928|R	>sp|Q9H0U4|RAB1B_HUMAN Ras-related protein	3	0	2	0	1	22.171	0	13.4	0	9229200
sp|Q9BZF9|UACA_HUMAN	>sp|Q9BZF9|UACA_HUMAN Uveal autoantigen w	1	3	5	3	5	162.5	2.9	3.9	0	48411000
sp|Q9Y3I0|RTCB_HUMAN	>sp|Q9Y3I0|RTCB_HUMAN RNA-splicing ligase	1	1	2	1	2	55.21	3.4	5	0	14165000
sp|Q13144|EI2BE_HUMAN	>sp|Q13144|EI2BE_HUMAN Translation initiation	1	0	1	0	1	80.379	0	1.1	0	16699000
sp|Q9Y4E5|ZN451_HUMAN	>sp|Q9Y4E5|ZN451_HUMAN E3 SUMO-protein l	1	1	1	1	1	121.48	0.7	0.7	0	27681000
sp|Q92841|DDX17_HUMAN	>sp|Q92841|DDX17_HUMAN Probable ATP-depe	1	1	4	0	1	80.272	1.6	6.3	0	18437000
sp|P08195|4F2_HUMAN	>sp|P08195|4F2_HUMAN 4F2 cell-surface antigen	1	0	2	0	2	67.993	0	4	0	13542000
sp|P06576|ATPB_HUMAN	>sp|P06576|ATPB_HUMAN ATP synthase subunit	1	0	2	0	2	56.559	0	4.5	0	12956000
sp|O00622|CCN1_HUMAN	>sp|O00622|CCN1_HUMAN CCN family member	1	0	1	0	1	42.026	0	2.6	0	11443000
sp|P28288|ABCD3_HUMAN	>sp|P28288|ABCD3_HUMAN ATP-binding cassett	1	0	3	0	3	75.475	0	4.2	0	16697000
sp|Q86YZ3|HORN_HUMAN;CON__Q86Y	>sp|Q86YZ3|HORN_HUMAN Hornerin OS=Homo	2	1	2	1	2	282.39	1.7	4.5	0	34201000
sp|P78385|KRT83_HUMAN;CON__Q6NT2	>sp|P78385|KRT83_HUMAN Keratin, type II cutic	3	1	13	0	0	54.195	1.4	30	0	12174000
sp|P38159|RBMX_HUMAN	>sp|P38159|RBMX_HUMAN RNA-binding motif p	1	0	1	0	1	42.331	0	3.3	0	9649600
sp|Q96ER3|SAAL1_HUMAN	>sp|Q96ER3|SAAL1_HUMAN Protein SAAL1 OS	1	0	1	0	1	53.557	0	2.1	0	8234100
sp|Q9NV70|EXOC1_HUMAN	>sp|Q9NV70|EXOC1_HUMAN Exocyst complex c	1	0	1	0	1	101.98	0	1.2	0	14565000
sp|P63261|ACTG_HUMAN	>sp|P63261|ACTG_HUMAN Actin, cytoplasmic 2 (	1	16	21	1	1	41.792	55.5	68.3	0	6093300
sp|Q9BUJ2|HNRL1_HUMAN	>sp|Q9BUJ2|HNRL1_HUMAN Heterogeneous nuc	1	1	1	1	1	95.737	1.6	1.6	0	10565000
sp|Q04695|K1C17_HUMAN;CON__Q0469	>sp|Q04695|K1C17_HUMAN Keratin, type I cytosk	2	8	12	0	1	48.105	17.1	25.5	0	8617700
sp|P10809|CH60_HUMAN	>sp|P10809|CH60_HUMAN 60 kDa heat shock pro	1	1	1	1	1	61.054	1.6	1.6	0	9418300
sp|O00571|DDX3X_HUMAN	>sp|O00571|DDX3X_HUMAN ATP-dependent RN	1	0	2	0	2	73.243	0	3.3	0	11160000
sp|Q92614|MY18A_HUMAN	>sp|Q92614|MY18A_HUMAN Unconventional my	1	1	1	1	1	233.11	0.7	0.7	0	27724000
sp|Q96A65|EXOC4_HUMAN	>sp|Q96A65|EXOC4_HUMAN Exocyst complex co	1	0	1	0	1	110.5	0	1.4	0	14580000
sp|Q13200|PSMD2_HUMAN	>sp|Q13200|PSMD2_HUMAN 26S proteasome nor	1	0	1	0	1	100.2	0	1.7	0	11765000
sp|Q5D862|FILA2_HUMAN;CON__Q5D86	>sp|Q5D862|FILA2_HUMAN Filaggrin-2 OS=Hom	2	1	1	1	1	248.07	0.5	0.5	0	17615000
sp|Q9BUQ8|DDX23_HUMAN	>sp|Q9BUQ8|DDX23_HUMAN Probable ATP-dep	1	1	1	1	1	95.581	1	1	0	10138000
sp|O94915|FRYL_HUMAN	>sp|O94915|FRYL_HUMAN Protein furry homolog	1	0	1	0	1	339.59	0	0.3	0	29315000
sp|Q5VWQ8|DAB2P_HUMAN	>sp|Q5VWQ8|DAB2P_HUMAN Disabled homolog	1	0	1	0	1	131.62	0	0.8	0	14583000
sp|Q9Y2F5|ICE1_HUMAN	>sp|Q9Y2F5|ICE1_HUMAN Little elongation comp	1	0	1	0	1	247.89	0	0.3	0	18734000
sp|Q00610|CLH1_HUMAN;sp|P53675|CLH	>sp|Q00610|CLH1_HUMAN Clathrin heavy chain	2	0	2	0	2	191.61	0	1.4	0	15131000
sp|P38646|GRP75_HUMAN	>sp|P38646|GRP75_HUMAN Stress-70 protein, mi	1	1	2	1	2	73.68	1.8	4	0	5838000
sp|P02786|TFR1_HUMAN	>sp|P02786|TFR1_HUMAN Transferrin receptor pr	1	0	1	0	1	84.87	0	1.8	0	5656900
sp|O60506|HNRPQ_HUMAN;sp|O43390|H	>sp|O60506|HNRPQ_HUMAN Heterogeneous nuc	2	1	1	1	1	69.602	1.9	1.3	0	4385900
sp|Q9Y224|RTRAF_HUMAN	>sp|Q9Y224|RTRAF_HUMAN RNA transcription,	1	0	1	0	1	28.068	0	5.7	0	2189400
sp|P35749|MYH11_HUMAN	>sp|P35749|MYH11_HUMAN Myosin-11 OS=Hon	1	11	11	0	1	227.34	6.6	7.3	0	12238000
sp|P78527|PRKDC_HUMAN	>sp|P78527|PRKDC_HUMAN DNA-dependent pro	1	0	4	0	4	469.08	0	1.2	0	21921000
sp|O75369|FLNB_HUMAN;sp|P21333|FLN	>sp|O75369|FLNB_HUMAN Filamin-B OS=Homo	3	0	1	0	1	278.16	0	0.4	0	3163400
sp|P63313|TYB10_HUMAN	>sp|P63313|TYB10_HUMAN Thymosin beta-10 OS	1	1	0	1	0	5.0256	36.4	0	0	0
sp|Q08554|DSC1_HUMAN	>sp|Q08554|DSC1_HUMAN Desmocollin-1 OS=H	1	1	0	1	0	99.986	1.7	0	0	0
sp|Q13177|PAK2_HUMAN	>sp|Q13177|PAK2_HUMAN Serine/threonine-prote	1	0	1	0	1	58.042	0	2.7	0	0
sp|Q9H4M9|EHD1_HUMAN;sp|Q9NZN3|E	>sp|Q9H4M9|EHD1_HUMAN EH domain-contain	3	0	1	0	1	60.626	0	1.7	0	0
sp|P05387|RLA2_HUMAN	>sp|P05387|RLA2_HUMAN 60S acidic ribosomal p	1	2	1	2	1	11.665	17.4	10.4	26859000	0
sp|P08729|K2C7_HUMAN;CON__Q3KNV	>sp|P08729|K2C7_HUMAN Keratin, type II cytosk	2	22	22	1	0	51.385	43.1	40.5	9453300	0
sp|P12956|XRCC6_HUMAN	>sp|P12956|XRCC6_HUMAN X-ray repair cross-co	1	3	1	3	1	69.842	6.2	1.6	47606000	0
sp|P16403|H12_HUMAN;sp|P10412|H14_H	>sp|P16403|H12_HUMAN Histone H1.2 OS=Homo	3	2	2	2	2	21.364	10.8	10.8	60044000	0
sp|P18124|RL7_HUMAN	>sp|P18124|RL7_HUMAN 60S ribosomal protein L	1	2	1	2	1	29.225	9.3	4.4	36784000	0
sp|P26599|PTBP1_HUMAN	>sp|P26599|PTBP1_HUMAN Polypyrimidine tract-	2	2	1	2	1	57.221	5.6	1.7	6198200	0
sp|P27694|RFA1_HUMAN	>sp|P27694|RFA1_HUMAN Replication protein A	1	1	0	1	0	68.137	2.1	0	14161000	0
sp|P36578|RL4_HUMAN	>sp|P36578|RL4_HUMAN 60S ribosomal protein L	1	1	1	1	1	47.697	2.6	2.8	18302000	0
sp|P36873|PP1G_HUMAN;sp|P62140|PP1B	>sp|P36873|PP1G_HUMAN Serine/threonine-prote	3	1	0	1	0	36.983	2.5	0	15506000	0
sp|P46781|RS9_HUMAN	>sp|P46781|RS9_HUMAN 40S ribosomal protein S	1	4	3	4	3	22.591	16	14.4	68049000	0
sp|P51116|FXR2_HUMAN	>sp|P51116|FXR2_HUMAN Fragile X mental retar	1	4	3	2	1	74.222	6.7	4.2	30822000	0
sp|P61513|RL37A_HUMAN;sp|A6NKH3|RI	>sp|P61513|RL37A_HUMAN 60S ribosomal protei	2	1	1	1	1	10.275	19.6	8.7	44225000	0
sp|P62263|RS14_HUMAN	>sp|P62263|RS14_HUMAN 40S ribosomal protein	1	2	2	2	2	16.273	9.3	15.9	60331000	0
sp|P62328|TYB4_HUMAN	>sp|P62328|TYB4_HUMAN Thymosin beta-4 OS=	1	1	0	1	0	5.0526	31.8	0	9606200	0
sp|P62805|H4_HUMAN	>sp|P62805|H4_HUMAN Histone H4 OS=Homo sa	1	3	3	3	3	11.367	29.1	29.1	64273000	0
sp|P62906|RL10A_HUMAN	>sp|P62906|RL10A_HUMAN 60S ribosomal protei	1	1	0	1	0	24.831	3.7	0	30802000	0
sp|P62917|RL8_HUMAN	>sp|P62917|RL8_HUMAN 60S ribosomal protein L	1	1	2	1	2	28.024	6.2	10.5	36336000	0
sp|P62995|TRA2B_HUMAN	>sp|P62995|TRA2B_HUMAN Transformer-2 protei	1	1	0	1	0	33.665	3.5	0	4844900	0
sp|P67809|YBOX1_HUMAN;sp|P16989|YB	>sp|P67809|YBOX1_HUMAN Y-box-binding prote	3	3	2	3	2	35.924	17.9	10.8	71022000	0
sp|Q00059|TFAM_HUMAN	>sp|Q00059|TFAM_HUMAN Transcription factor A	1	4	1	4	1	29.096	15.9	3.3	115000000	0

sp|Q00839|HNRPU_HUMAN	>sp|Q00839|HNRPU_HUMAN Heterogeneous nuc 1	2	1	2	1	90.583	1.9	0.8	39579000	0
sp|Q01130|SRSF2_HUMAN;sp|Q9BRL6|SR	>sp|Q01130|SRSF2_HUMAN Serine/arginine-rich : 2	2	1	2	1	25.476	6.8	3.6	161370000	0
sp|Q08211|DHX9_HUMAN	>sp|Q08211|DHX9_HUMAN ATP-dependent RNA 1	2	1	2	1	140.96	1.7	0.8	32109000	0
sp|Q13247|SRSF6_HUMAN	>sp|Q13247|SRSF6_HUMAN Serine/arginine-rich : 3	2	2	2	2	39.586	5.8	5.2	31182000	0
sp|Q14444|CAPR1_HUMAN	>sp|Q14444|CAPR1_HUMAN Caprin-1 OS=Homo 1	2	1	2	1	78.365	2.8	1.6	65844000	0
sp|Q15717|ELAV1_HUMAN	>sp|Q15717|ELAV1_HUMAN ELAV-like protein 1 1	2	2	2	2	36.091	5.5	7.4	43383000	0
sp|Q16629|SRSF7_HUMAN	>sp|Q16629|SRSF7_HUMAN Serine/arginine-rich : 1	2	0	2	0	27.366	7.1	0	49597000	0
sp|Q5TZA2|CROCC_HUMAN	>sp|Q5TZA2|CROCC_HUMAN Rootletin OS=Hor 1	1	0	1	0	228.52	0.4	0	19528000	0
sp|Q7Z406|MYH14_HUMAN	>sp|Q7Z406|MYH14_HUMAN Myosin-14 OS=Ho 1	8	8	2	1	227.87	4.7	4.1	265350000	0
sp|Q7Z417|NUFP2_HUMAN	>sp|Q7Z417|NUFP2_HUMAN Nuclear fragile X m 1	2	0	2	0	76.12	3.9	0	32654000	0
sp|Q8IYS0|ASTRC_HUMAN	>sp|Q8IYS0|ASTRC_HUMAN Protein Aster-C OS 1	1	0	1	0	76.034	1.1	0	33941000	0
sp|Q8ND56|LS14A_HUMAN	>sp|Q8ND56|LS14A_HUMAN Protein LSM14 hon 1	1	0	1	0	50.529	2.2	0	11350000	0
sp|Q8TA86|RP9_HUMAN	>sp|Q8TA86|RP9_HUMAN Retinitis pigmentosa 9 1	1	1	1	1	26.106	4.1	6.8	16501000	0
sp|Q969Q0|RL36L_HUMAN;sp|P83881|RL3	>sp|Q969Q0|RL36L_HUMAN 60S ribosomal prote 2	2	1	2	1	12.469	16	8.5	105780000	0
sp|Q96PK6|RBM14_HUMAN	>sp|Q96PK6|RBM14_HUMAN RNA-binding prote 1	2	0	2	0	69.491	3.7	0	34896000	0
sp|Q99880|H2B1L_HUMAN;sp|Q99879|H2l	>sp|Q99880|H2B1L_HUMAN Histone H2B type 1- 15	2	1	2	1	13.952	15.1	5.6	19512000	0
sp|Q9P0W8|SPAT7_HUMAN	>sp|Q9P0W8|SPAT7_HUMAN Spermatogenesis-as 1	1	0	1	0	67.718	1.2	0	127400000	0
sp|Q9Y2C9|TLR6_HUMAN	>sp|Q9Y2C9|TLR6_HUMAN Toll-like receptor 6 C 1	1	0	1	0	91.879	0.9	0	70854000	0
