# Supplementary material for: FOXD1-dependent RalA-ANXA2-Src complex promotes CTC formation in breast cancer
Source: J Exp Clin Cancer Res. 2022 Oct 13;41:301. doi: 10.1186/s13046-022-02504-0 (PMC9558416; doi:10.1186/s13046-022-02504-0)
Supplement: Supplementary file 4 — Additional file 4: Supplementary Table 1. List of top 5 candidates of RalA-interacting proteins that were identified by co-immunoprecipitation and MS. Supplementary Table 2. ZDOCK used to predict the binding sites of RalA and ANXA2. Supplementary Table 3. The sequences of the primers. Supplementary Table 4. The antibodies applied in this study. [file 13046_2022_2504_MOESM4_ESM.pdf]

Additional file 4. Supplementary tables.

Gene Name	Unique peptides	LFQ intensity	Coverage [%]	Molecular Weight [kDa]	Accession
Importin-5	36	3.34E+09	45.1	123.63	O00410
Importin subunit beta-1	18	1.00E+09	26.5	97.169	Q14974
Annexin A2	7	2.96E+08	25.7	38.604	P07355
Importin-7	20	6.80E+08	23.7	119.52	O95373
Importin-9	12	3.05 E+08	13.7	115.96	Q96P70

Supplementary Table 1. List of top 5 candidates of RalA-interacting proteins that were identified by co-immunoprecipitation and MS.

Types	Distance	From	To
**Salt Bridge**	2.74436	ANNEXIN A2:ARG37:NH2	RALA:ASP133:OD2
**Salt Bridge**	3.09922	ANNEXIN A2:ARG304:NH1	RALA:GLU141:OE2
**Salt Bridge**	3.19641	ANNEXIN A2:LYS313:NZ	RALA:ASP106:OD1
**Salt Bridge**	3.87825	ANNEXIN A2:LYS313:NZ	RALA:GLU109:OE1
**Salt Bridge**	3.28976	RALA:LYS134:NZ	ANNEXIN A2:ASP52:OD1
**Attractive Charge**	3.07264	ANNEXIN A2:LYS329:NZ	RALA:GLU99:OE1
**Attractive Charge**	2.3048	RALA:ARG145:NH1	ANNEXIN A2:ASP338:OD1
**Attractive Charge**	4.00128	RALA:ARG145:NH2	ANNEXIN A2:ASP338:OD2
**Attractive Charge**	5.48941	RALA:ARG145:NH2	ANNEXIN A2:ASP339:OD1
**Conventional Hydrogen Bond**	3.3009	ANNEXIN A2:ASN41:ND2	RALA:ASP133:OD1
**Carbon Hydrogen Bond**	3.72504	RALA:SER138:CB	ANNEXIN A2:ASN62:OD1
**Pi-Sulfur**	4.74359	RALA:MET98:SD	ANNEXIN A2:TYR316
**Alkyl**	5.32987	ANNEXIN A2:LEU332	RALA:MET98
**Pi-Alkyl**	3.99311	ANNEXIN A2:TYR333	RALA:MET98

Supplementary Table 2. ZDOCK used to predict the binding sites of RalA and ANXA2.

Gene	Sequence (5' to 3')
**Real time RT-PCR primers**	
FOXD1-F	TGAGCACTGAGATGTCCGATG
FOXD1-R	CACCACGTCGATGTCTGTTTC
RalA-F	ATGGCTGCAAATAAGCCCAAG
RalA-R	TGTCTGCTTTGGTAGGCTCATA
GAPDH-F	CATGGGTGTGAACCATGAGA
GAPDH-R	GTCTTCTGGGTGGCAGTGAT
**shRNA target sequences**	
shFOXD1-1	tgTCCAGTGTCGAGAACTTTA
shFOXD1-2	gcCCTTCTCCATCGAGAGCAT
shFOXD1-3	ccTCGCCGAGGAAACAGACAT
**siRNA target sequences**	
si-RalA-1	GGACTACGCTGCAATTAGA
si-RalA-2	GGAAGAAGGTAGTGCTAGA
si-RalA-3	AGATGAGAATGTTCCATTT
si-ANXA2-1	ACCAGCTTGCGAATAACAG
si-ANXA2-2	TTGACCAACCGCAGCAATG
si-ANXA2-3	TTGCTGATCGGCTGTATGA
si-Src-1	GTTGTATGCTGTGGTTTCA
si-Src-2	CTCGGCTCATTGAAGACAA
si-Src-3	GAGAGAACCTGGTGTGCAA
**ChIP-qPCR primers**	
RalA-Fragment3-F	CATGATCACCCTCCTCTCAG

RalA-Fragment3-R	CCACAGATCACTTCCGG

Supplementary Table 3. The sequences of the primers.

Antibody	Company	Catalog no.	Dilution
**Western blotting**			
FOXD1	Abcam	ab49156	1/2000
E-cadherin	CST	3195	1/1000
N-cadherin	CST	13116	1/1000
Vimentin	CST	5741	1/1000
Snail	CST	3879	1/1000
GAPDH	Proteintech	10494-1-AP	1/5000
RalA	Proteintech	13629-1-AP	1/1500
p-mek1/2	CST	9154	1/1000
mek1/2	CST	9122	1/1000
p-erk1/2	CST	4370	1/2000
erk1/2	CST	4695	1/1000
β-catenin	CST	8480	1/1000
Src	CST	2109	1/1000
p-ANXA2 (Tyr23)	Santa Cruz	sc-135753	1/500
ANXA2	Abclonal	A12397	1/1000
Mouse	Abcam	ab6789	1/5000
Rabbit	Abcam	ab6721	1/5000
HA-Tag antibody	CST	3724	1/1000
HRP-conjugated Goat Anti-Mouse IgG Heavy Chain	Abclonal	AS064	1/5000
HRP-conjugated Goat Anti-Mouse IgG Light Chain	Abclonal	AS062	1/5000
**Immunohistochemistry**			
FOXD1	Abcam	ab179940	1/150

E-cadherin	CST	3195	1/400
Vimentin	CST	5741	1/100
p-erk1/2	CST	4370	1/200
p-mek12	CST	2338	1/100
RalA	Proteintech	13629-1-AP	1/300
**Immunofluorescence**			
E-cadherin	CST	14472	1/100
Vimentin	CST	5741	1/100
ANXA2	Abclonal	A12397	1/100
p-ANXA2 (Tyr23)	Santa Cruz	sc-135753	1/50
RalA	Huabio	EM1707-77	1/50
RalA	Abclonal	A11736	1/50
Anti-mouse IgG (H+L), F(ab')2 Fragment (Alexa Fluor® 647 Conjugate)	CST	4410	1/1000
Goat Anti-Rabbit IgG - H&L (Alexa Fluor® 647)	Abcam	Ab150079	1/2000
Goat Anti-Mouse IgG - H&L (Alexa Fluor® 555)	Abcam	Ab150114	1/2000
Goat Anti-Rabbit IgG H&L (Alexa Fluor® 555)	Abcam	Ab150078	1/2000
DAPI	Solarbio	C0060	1:100
Mounting Medium, antifading	Solarbio	S2100	
**Chromatin Immunoprecipitation**			

FOXD1	Santa Cruz	sc-293238	5.0 ug
Mouse IgG Isotype Control	Invitrogen	31903	5.0 ug
**CUT&Tag**			
FOXD1	Santa Cruz	sc-293238	1/50
Mouse IgG Isotype Control	Invitrogen	31903	1/50
NovoNGS® CUT&Tag 2.0 High-Sensitivity Kit	Novoprotein	N259-YH01	
**GST pull-down assay**			
Anti-GST Tag Magnetic Beads	Sino Biological	TB11213	
GST Tag Polyclonal antibody	Proteintech	10000-0-AP	1:2000
His-Tag Monoclonal antibody	Proteintech	66005-1-Ig	1:5000
**Co-immunoprecipitation (Co-IP)**			
Anti-FLAG antibody	Sigma-Aldrich	F1804	
Anti-ANXA2 antibody	Santa cruz	Sc-28385	
Anti-Src antibody	Santa cruz	Sc-8056	
Mouse IgG Isotype Control	Invitrogen	31903	
Pierce™Classic Magnetic IP/Co-IP Kit	ThermoFisher	88804	

Supplementary Table 4. The antibodies applied in this study.
